# The cell–cell junctions of mammalian testes: I. The adhering junctions of the seminiferous epithelium represent special differentiation structures

**DOI:** 10.1007/s00441-014-1906-9

**Published:** 2014-06-08

**Authors:** Lisa M. Domke, Steffen Rickelt, Yvette Dörflinger, Caecilia Kuhn, Stefanie Winter-Simanowski, Ralf Zimbelmann, Rina Rosin-Arbesfeld, Hans Heid, Werner W. Franke

**Affiliations:** 1 Helmholtz Group for Cell Biology, German Cancer Research Center (DKFZ), Im Neuenheimer Feld 280, 69120 Heidelberg, Germany; 2 Brandenburg University of Technology, Senftenberg, Germany; 3 Progen Biotechnik GmbH, Heidelberg, Germany; 4 Department of Anatomy, Sackler School of Medicine, Tel-Aviv University, Tel-Aviv, Israel; 5 Present Address: Whitehead Institute for Biomedical Research, Massachusetts Institute of Technology (MIT), Cambridge, MA USA; 6 Present Address: David H. Koch Institute for Integrative Cancer Research, Massachusetts Institute of Technology (MIT), Cambridge, MA USA

**Keywords:** Adherens junction, Desmosomes, Sertoli cells, Seminiferous tubules, *Areae adhaerentes*, Cribelliform junctions

## Abstract

The seminiferous tubules and the excurrent ducts of the mammalian testis are physiologically separated from the mesenchymal tissues and the blood and lymph system by a special structural barrier to paracellular translocations of molecules and particles: the “blood–testis barrier”, formed by junctions connecting Sertoli cells with each other and with spermatogonial cells. In combined biochemical as well as light and electron microscopical studies we systematically determine the molecules located in the adhering junctions of adult mammalian (human, bovine, porcine, murine, i.e., rat and mouse) testis. We show that the seminiferous epithelium does not contain desmosomes, or “desmosome-like” junctions, nor any of the desmosome-specific marker molecules and that the adhering junctions of tubules and ductules are fundamentally different. While the ductules contain classical epithelial cell layers with E-cadherin-based adherens junctions (AJs) and typical desmosomes, the Sertoli cells of the tubules lack desmosomes and “desmosome-like” junctions but are connected by morphologically different forms of AJs. These junctions are based on N-cadherin anchored in cytoplasmic plaques, which in some subforms appear thick and dense but in other subforms contain only scarce and loosely arranged plaque structures formed by α- and β-catenin, proteins p120, p0071 and plakoglobin, together with a member of the striatin family and also, in rodents, the proteins ZO-1 and myozap. These N-cadherin-based AJs also include two novel types of junctions: the “*areae adhaerentes*”, i.e., variously-sized, often very large cell-cell contacts and small sieve-plate-like AJs perforated by cytoplasm-to-cytoplasm channels of 5–7 nm internal diameter (“cribelliform junctions”). We emphasize the unique character of this epithelium that totally lacks major epithelial marker molecules and structures such as keratin filaments and desmosomal elements as well as EpCAM- and PERP-containing junctions. We also discuss the nature, development and possible functions of these junctions.

**Electronic supplementary material:**

The online version of this article (doi:10.1007/s00441-014-1906-9) contains supplementary material, which is available to authorized users.

## Introduction

A unique type of epithelium-like tissue element, in which somatic cell proliferation and differentiations occur side by side with meiotic divisions and male germ cell differentiation (spermatogenesis), is the *tubulus seminiferus* of the mammalian testis. Here, basal lamina-founded somatic cells, the “Sertoli cells”, are laterally connected to each other and to spermatogenic cells with multiple cell-to-cell attachment structures (Dym and Fawcett 1970; Dym 1977; Russell and Peterson 1985; Pelletier 2001). Moreover, the Sertoli and the germ cells form an obviously tight-fitting barrier for paracellular translocations of molecules and particles, the tight junction-based blood–testis barrier (BTB) and support the development of the germ cells, at least up to the point of spermatid differentiation, in specific Sertoli cell indentations (“pockets”) harboring the spermatid heads (e.g., Dym 1977; Vogl et al. 1991, 2008, 2013; Southwood and Gow 2001; Wong and Cheng 2005). Although *prima facie* the mature Sertoli cell layer looks like a typical epithelium, these cells are profoundly different from all other epithelial cells with respect to their biochemical and morphological components as well as their general architecture. This holds in particular for the absence of intermediate-sized filaments (IFs) of the keratin type, for the presence of vimentin IFs (Franke et al. 1979; see also Spruill et al. 1983; Paranko and Virtanen 1986; Franke et al. 1989; Stosiek et al. 1990; Steger and Wrobel 1994; Steger et al. 1994), for the additional occurrence of neurofilaments in human Sertoli cells (see, e.g., Davidoff et al. 1999) and for the presence of various types of specific adherens junctions (AJs) between the Sertoli cells (homotypic) and between Sertoli cells and spermatogonial cells in the basal part of the Sertoli cells (heterotypic-basolateral junctions) and between the adluminal pockets of the Sertoli cells and the spermatid heads (heterotypic-apical junctions).

Originally, in the early years of transmission electron microscopy, certain AJs connecting Sertoli cells with each other or with spermatogonial cells had been seen as typical desmosomes or as desmosome-related and thus classified as “desmosomes”, “rudimentary desmosomes”, or “desmosome-like junctions” (e.g., Nicander 1967; Altorfer et al. 1974; Russell 1977a, b, c; Connell 1978; Nagano and Suzuki 1978; Osman 1978; Osman and Plöen 1978). Although our laboratory has repeatedly reported the total absence of both specific desmosomal structures and desmosomal marker molecules from Sertoli cells of the mature mammalian testis for more than three decades (e.g., Franke et al. 1979, 1981, 1982, 1983, 1986; 1989; Mueller and Franke 1983; Moll et al. 1986; Schmelz et al. 1986; Theis et al. 1993; see also Pelletier and Byers 1992; Schäfer et al. 1994; Nuber et al. 1995; Mertens et al. 1996), other authors have claimed, again and again, the occurrence of desmosomes or “desmosome-like” junctions in Sertoli cells of mature mammals active in spermatogenesis (Vogl et al. 2008; Li et al. 2009; Lie et al. 2010, 2011; Cheng et al. 2011; Mruk and Cheng 2011; see Table 1 and Electronic Supplementary Material, Table S1). Because of this long and still ongoing controversy, the potential diagnostic value of molecular markers in histology and pathology and also in view of the worldwide interest in the development of male contraceptive agents based on the interference with cell–cell interactions in the testis (e.g., O’Donnell et al. 2000; Cheng and Mruk 2002, 2011, 2012; Lee and Cheng 2004; Mruk and Cheng 2004a, b; Wong et al. 2005; Xia et al. 2005; Lee et al. 2009; Mok et al. 2012, 2013a, b), we decided to study the cell biology of the *tubuli seminiferi* of diverse mammalian species. We were particularly interested in the connections and interactions of Sertoli cells with each other and with the spermatogonial cells. Therefore, we analyzed these interactions in ultrastructural and molecular biological detail using the epithelium of the excurrent duct system as parallel controls. The results concerning the adhering junctions of the *tubuli seminiferi* are presented in this report. Results on gap and tight junctions, other cell–cell adhesion structures, the junctions in the peritubular and interstitial cells of the testis, junctional and cytoskeletal structures identified in early developmental stages, in aged and pathological forms, and in cultured Sertoli cells will be dealt with in further publications.

**Table 1:** Reports claiming that desmosomes or desmosome-like junctions or desmosome-specific molecules occur in the *tubuli seminiferi* of mammalian testes (only references since 1983 are considered here, as identifications using molecule-specific antibodies against desmosomal components have been generally available since that year); for complete references, see Electronic Supplementary Material, Table S1

Alves et al. (2013)
Bergmann et al. (1984)
Chapin et al. (2001)
Cheng and Mruk (2002, 2011, 2012)
Cheng et al. (2011, 2013)
Goossens and van Roy (2005)
Johnson and Boekelheide (2002a,b)
Kopera et al. (2010)
Lee and Cheng (2004)
Lee et al. (2009)
Li JCH et al. (2001)
Li MWM et al. (2009, 2010)
Lie et al. (2010, 2011)
Mok et al. (2013a)
Morrow et al. (2010)
Mruk and Cheng (2004a, b, 2011)
Mruk et al. (2013)
Mullholland et al. (2001)
Russell and Peterson (1985)
Su et al. (2013)
Vogl et al. (2000, 2008, 2013a,b)
Wine and Chapin (1999)
Wong and Cheng (2005)
Wong et al. (2004, 2005)
Xia et al. (2005)
Yan et al. (2007)
Yan and Cheng (2005)
Zhang et al. (2005a,b)

## Materials and methods

### Tissues and cells

Bovine testicular tissue samples were obtained in the regional slaughterhouse (Mannheim, Germany). Murine (rat and mouse) testes from sexually mature animals were obtained from the Central Animal Laboratory of the German Cancer Research Center (Heidelberg, Germany; for details, see Franke et al. 2006, 2013). In addition, testis tissue specimens from 3-year-old boars of German landrace pigs were provided by the Institute of Farm Animal Genetics (Friedrich-Loeffler-Institute, Mariensee, Germany; cf. Rickelt et al. 2011). Cryopreserved and aldehyde-fixed human testis samples were obtained from surgical material taken, examined for diagnostic pathology and processed in compliance with the regulations of the Ethics Committees of the Universities of Heidelberg and Marburg (Germany; see also Langbein et al. 2003).

In general, the tissue samples were fixed at ca. 4 °C or at room temperature in most cases with 4 % formaldehyde, freshly prepared from paraformaldehyde, or in phosphate-buffered saline (PBS), with or without millimolar additions of divalent cations and embedded in paraffin. Alternatively, the tissues was snap-frozen in isopentane that had been precooled in liquid nitrogen and then stored at −80 °C until use (see references cited above). Suitable frozen tissue samples were used for preparations of “semi-thin” cryostat sections (ca. 5–15 μm thick), or thin sections for immunofluorescence microscopy (see below) and analyses by SDS-polyacrylamide gel electrophoresis (SDS-PAGE) of polypeptides and immunoblotting (see below).

### Antibodies

The antibodies (Abs) used in immunofluorescence and immunoelectron microscopy or in immunoblotting analyses of gel-electrophoretically-separated polypeptides are listed in Supplementary Table S2. Antigen-bound primary Abs were visualized with goat anti-rabbit, goat anti-guinea pig, or goat anti-mouse IgG (H+L) secondary Abs coupled to Cy3 (Dianova, Hamburg, Germany) or Alexa 488 (MoBiTec, Göttingen, Germany). For immunoblot analysis, horseradish-peroxidase-conjugated secondary Abs were used (Dianova).

### Gel electrophoresis and immunoblotting

Proteins of tissue samples, including microdissected ones, were analyzed by SDS-PAGE, followed by immunoblotting, as previously described (Rickelt et al. 2010, 2011; Straub et al. 2011; Pieperhoff et al. 2012). Usually 100–200 cryostat sections of ca. 5–15 μm thickness were collected and proteins of tissue lysates were solubilized in the same sample buffer (cf. Pieperhoff et al. 2012; Franke et al. 2013). After homogenization, the tissue lysates were heated to—and kept at—ca. 95 °C for 4–5 min, then centrifuged at 15,000 *g* for 10 min. Both the supernatant and the pelleted proteins and glycoproteins were subjected to SDS-PAGE, followed by transfer to polyvinylidene difluoride membranes (ImmobilonP; Millipore, Bedford, MA, USA). For immunoblot analyses, horseradish-peroxidase-conjugated secondary Abs were applied in combination with an enhanced chemiluminescence system (ECL; Fisher Scientific, Schwerte, Germany).

### Immunofluorescence microscopy

The methods used for immunofluorescence microscopy have been described (Langbein et al. 2003; Franke et al. 2006, 2013, 2014; Rickelt et al. 2008, 2011; Pieperhoff et al. 2012; Rickelt 2012). The frozen tissue sections were mounted on coverslips, air-dried and fixed for 5 or 10 min in −20 °C acetone. The specimens were rehydrated in PBS and pre-incubated in PBS containing 0.2 or 0.3 % Triton X-100 for 5 min before application of the primary Abs. In parallel, the tissue samples were fixed for 5–7 min in PBS (pH 7.4) containing 2 % formaldehyde, freshly prepared from paraformaldehyde and the cells were permeabilized with PBS containing 0.1 or 0.2 % saponin (same pH value; 5 min), followed by exposure to the specific primary Abs in PBS for 1 h. Following several washes in PBS for 5–10 min each, the samples were exposed to the specific secondary Abs for 30 min. After two or three washes for 5–10 min in PBS, the cell preparations or cryosections were rinsed in distilled water, fixed for 1 or 5 min in ethanol and mounted in Fluoromount G (Southern Biotech; obtained through Biozol Diagnostica, Eching, Germany). Finally, immunofluorescence microscopic images were recorded with an Axiophot II photomicroscope (Carl Zeiss, Jena, Germany), equipped with an AxioCam HR (Carl Zeiss). For confocal laser scanning microscopy, a Zeiss LSM 510 Meta instrument was used.

### Electron and immunoelectron microscopy

The electron and immunoelectron microscopy protocols were essentially as described (see Langbein et al. 2003; Franke et al. 2006, 2013; Rickelt et al. 2008, 2011). For immunoelectron microscopy of cryostat sections, the tissue samples were fixed in PBS containing 2 or 3 % formaldehyde for 5–7 min and permeabilized with PBS containing 0.1 % saponin (3–5 min), followed by incubation with primary Abs for at least 2 h. After three washing steps, the samples were incubated with secondary Abs conjugated with 1.4-nm gold particles (Nanogold; Biotrend, Cologne, Germany) for 4 h, followed by silver enhancement for various periods of time (5, 7, or 9 min). Electron micrographs were taken at 80 kV in an EM 900 or EM 10 (Carl Zeiss) instrument.

## Results

### For comparison and control: the epithelia of the excurrent ducts

In all species examined, we used tissue samples from various portions of the excurrent ducts, which are known to contain typical simple or columnar epithelia, as controls of the methods applied and to determine the specificity of the antibodies used in immunocytochemistry of the seminiferous tubules (for references, see, e.g., Dym 1974, 1976, 1977; Kasper and Stosiek 1989; Cyr et al. 1995, 2007; Piomboni 1997; Pelletier 2001; DeBellefeuille et al. 2003). Frequently, it was possible for us to study testicular tissue containing seminiferous tubules in parallel with preparations of excurrent duct tissues, including the epididymis, from the same animal.

### Biochemical results

**Fig. 1:**
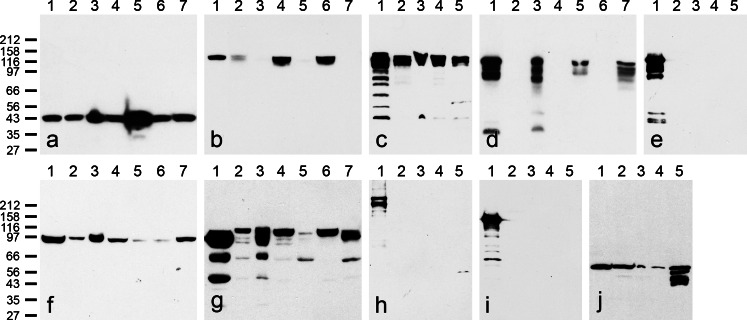
Identification of proteins and glycoproteins of cell-cell adhering junctions in dissected tissue parts of mammalian testis, enriched in seminiferous tubules, or excurrent duct tissues, demonstrated after SDS-PAGE of total protein lysates by immunoblotting with specific antibodies **a** Antibodies against β-actin identify a single polypeptide band at 43 kDa in cultured human HaCaT keratinocytes (*lane 1* in all blots shown, i.e. **a**–**j**), bovine testis (*lane 2*) and epididymis (*lane 3*), boar testis (*lane 4*) and boar excurrent duct-containing tissue (*lane 5*), rat testis (*lane 6*) and rat excurrent duct tissue (*lane 7*). **b** Antibodies against N-cadherin recognize this glycoprotein in testicular tissue of bull (*lane 2*), boar (*lane 4*) and rat (*lane 6*) but not to a significant extent in excurrent duct tissues of bovine (*lane 3*), porcine (*lane 5*) and murine (rat, *lane 7*) origin. **c** As in (**b**), N-cadherin is detected to comparable intensities in near-equal amounts of testicular tissue of bovine (*lane 2*), rat (*lane 3*), mouse (*lane 4*) and human (*lane 5*) origin. **d** In a mutually exclusive way, E-cadherin is recognized in the excurrent duct tissues of bovine (*lane 3*), porcine (*lane 5*) and murine (rat, *lane 7*) origin but not in the corresponding testicular tissues enriched in seminiferous tubules (material in *lanes 2*, *4* and *6* of bovine, porcine, and murine origin). **e** E-cadherin is not detected in tissue material from microdissected testicular tissues enriched in seminiferous tubules of bovine (*lane* 2), rat (*lane* 3), mouse (*lane* 4) and human (*lane* 5) origin. **f** The major plaque protein of AJs, β-catenin, is detected in all these tissues (*lanes 2*–*7* as in **a**, **b** and **d**), although in different intensities (note the weak reaction in *lanes 5* and *6*). **g** Similarly, the AJ plaque protein p120 is found in all samples, although at a rather low intensity in boar excurrent duct tissues (*lane 5*; note, however, a major proteolytic degradation product of about 62 kDa in *lanes 3*, *5* and *7*). **h** In comparison with human HaCaT keratinocytes (*lane 1*), desmoplakin is not recognized in bovine (*lane 2*), rat (*lane 3*), mouse (*lane 4*,) and human (*lane 5*) seminiferous tubules of testis. **i** Correspondingly, desmoglein Dsg-2 (HaCaT cells, *lane 1*) is not detected in bovine (*lane 2*), rat (*lane 3*), mouse (*lane 4*) and human (*lane 5*) testicular tissue containing seminiferous tubules. **j** Control showing the presence of vimentin in all tissues shown in (**h–j**)

When we compared the adhering junction proteins of specific intercepts or portions of the excurrent system with those of the seminiferous tubule system by gel electrophoresis and immunoblot identification, we observed striking differences (Fig. 1). Certain cytoskeletal control proteins such as actin and vimentin (Fig. 1a, j), as well as constituents known to occur in all kinds of AJs such as the *armadillo*-type plaque proteins β-catenin, protein p120 and plakoglobin, which is known also to occur in desmosomes (Cowin et al. 1986), were detected in all samples (e.g., Fig. 1f, g). Other molecules showed marked differences between the AJs of the excurrent duct and the seminiferous tubule epithelia. In all species examined, E-cadherin was found as a major component in all excurrent duct regions but was not detected in the semini[-]ferousrous epithelium (e.g., Fig. 1d, e; for a special minor non-epithelial cell type, see below). Vice versa, N-cadherin was present as a major component in the seminiferous tubule epithelia but was absent in the excurrent duct epithelia (e.g., Fig. 1b, c). No other AJ cadherins examined were found in either kind of epithelia, while the AJ plaque proteins, α- and β-catenin, p120, p0071, myozap and a member of the striatin family, were abundantly present in both excurrent duct and seminiferous tubule epithelia (see Table 2 for specific comments).

**Table 2:** Homotypic adhering junctions: results of immunolocalization experiments (summary for all species examined)

Molecule	Sertoli–Sertoli and Sertoli–Spermatogonia cell junctions	Excurrent duct epithelia
Transmembrane glycoproteins (Cadherins)		
E-cadherin	−	+
VE-cadherin	−	−
N-cadherin	+	−
P-cadherin	−	−
Cadherin-11	−	−
Desmoglein 1 (Dsg-1)	−	−
Desmoglein 2 (Dsg-2)	−	+
Desmoglein 3 (Dsg-3)	−	−
Desmocollin 1 (Dsc-1)	−	−
Desmocollin 2 (Dsc-2)	−	+
Desmocollin 3 (Dsc-3)	−	−
Other transmembrane molecules		
EpCAM	−	+
Protein PERP	−	+
Cytoplasmic plaque proteins		
Desmoplakin I+II	−	+
Plakophilin 1	−	−
Plakophilin 2	−	+
Plakophilin 3	−	+
β-Catenin	+	+
Protein p120	+	+
Protein p0071	+	+
Plakoglobin	+	+
Neurojungin	−	−
α-Catenin	+	+
Protein ZO-1	(+/−)^a^	+
Myozap	(+/−)^a^	+
Striatin^b^	+	+
Protein LUMA	–	+

^a^For unexplained reasons, we have consistently found reactions of the plaque proteins myozap and ZO-1 in adherens junctions of Sertoli cells in the rodent testes but not in the other species examined in which only the adherens junctions of the endothelial and some interstitial mesenchymal cells showed myozap- and/or ZO-1-positive junctions

^b^Whether it is striatin or a closely related member of the striatin family of proteins is not yet clear

The results for desmosomal proteins of both kinds were very impressive and clear: As a representative example of the desmosomal cadherins, the absence of desmoglein Dsg-2 in the seminiferous tubules is shown in Fig. 1i and the absence of the plaque protein desmoplakin in Fig. 1h. Negative results in the seminiferous tubules were also obtained for desmocollin Dsc-2 and plakophilin Pkp-2 as well as molecules Dsg-1 and Dsg-3, Dsc-1 and Dsc-3 and Pkp-1 and Pkp-3 (not shown). In contrast, certain desmosomal molecules (Dsc-2, Dsg-2, Pkp-2 and desmoplakin) were abundantly present in all the epithelia of the excurrent duct system (Table 2).

The transmembrane proteins EpCAM and PERP, which were not detected in the seminiferous epithelia, were regular constituents of the subapical region close to the *zonula adhaerens* AJs of the excurrent ducts (these molecules will be specifically dealt with in one of the next publications in this series).

### Immunofluorescence microscopy results

#### Excurrent duct epithelia

**Fig. 2:**
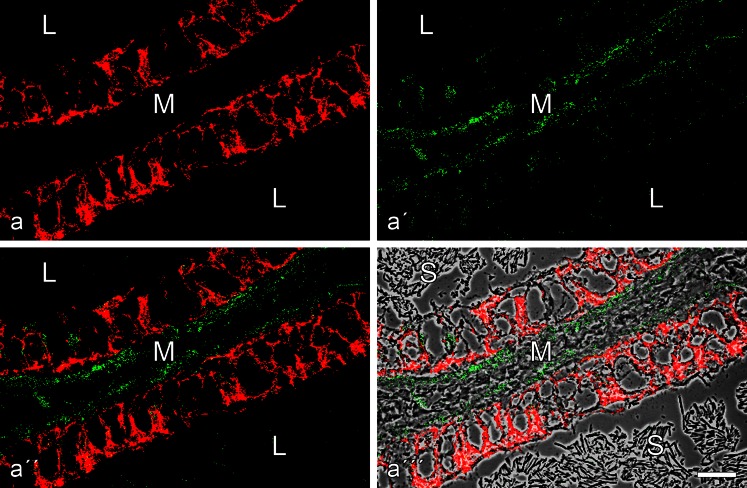
Double-label immunofluorescence microscopy of a near-longitudinal cryostat section through frozen rat testis tissue containing excurrent ducts (*L* lumen; *M* mesenchymal space with interstitial cells) after reactions with antibodies to E-cadherin (**a**, *red*) or N-cadherin (**a'**, *green*), demonstrating the mutually exclusive localization of both cadherins (**a''**; **a'''** with phase contrast background). Note the extensive and intensive E-cadherin reaction along the plasma membranes of the ductal epithelium (**a**, **a''**) as well as the weak reaction of N-cadherin in some of the interstitial cells of the mesenchyme (*green* in **a'**). Note that the ductal lumen is filled with masses of aggregated spermatozoa (*S* in **a'''**). *Bar* 20 μm

**Fig. 3:**
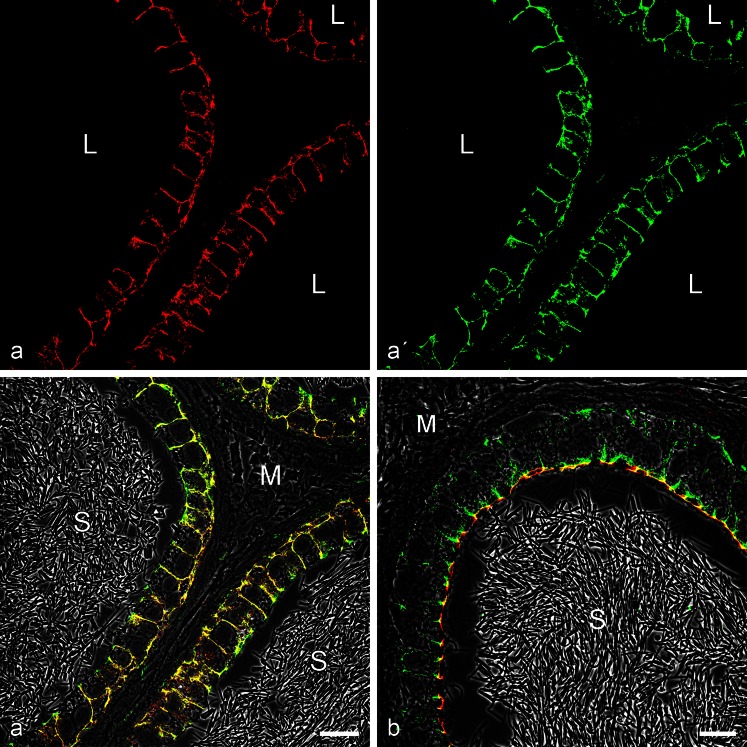
This double-label immunofluorescence microscopy shows the specific immunostaining of the AJs connecting excurrent duct epithelial cells of rat testis after reactions with the *armadillo* plaque proteins p120 (**a**, *red*, mouse mAb) and β-catenin (**a'**, *green*, rabbit antibodies), resulting in colocalization indicated by the *yellow* merger staining (**a''**, on a phase contrast background) in the subapical *zonula adhaerens* as well as in the numerous AJs along the lateral membrane-membrane contacts. By contrast in (**b**), colocalization of β-catenin with the non-*armadillo* plaque protein, myozap (*red*, mouse mAb), indicates that in this case protein myozap also occurs in the subapical *zonula* but is not detectable in significant amounts in most of the lateral membrane junctions. *L* lumen; *M* mesenchymal region; *S* aggregates of spermatozoa. *Bar* 20 μm

The results revealed in all the epithelia of the excurrent duct system a keratin IF cytoskeleton and an abundance of desmosomes that could generally be demonstrated by co-immunolocalization of the corresponding transmembrane and the cytoplasmic plaque molecules (Table 2; an example for desmoglein Dsg-2 and desmoplakin is shown in Electronic Supplementary Material, Fig. S1). By contrast, differential localization of E-cadherin and N-cadherin is seen in the tissues in which N-cadherin was selectively absent in the epithelia and only seen as a very minor element in some of the interstitial cells (Fig. 2). As to the cytoplasmic plaque proteins of the epithelial AJs, we noted that some occurred in all junction structures, i.e., *zonula adhaerens* plus *fasciae adhaerentes* and *puncta adhaerentia*, such as the catenins and protein p120 (Fig. 3a–a''), whereas others such as proteins myozap, p0071 and a member of the striatin family appeared to be largely restricted to the subapical *zonula adhaerens* (see, e.g., Fig. 3b). Protein PERP was a particularly prominent component associated with the *zonula adhaerens* structure (not shown; for comparison with other epithelia, see Franke et al. 2013) and specific *zonula* immunolocalizations were also noted with some of the antibodies directed against protein LUMA (cf. Franke et al. 2014). The massive aggregates of spermatozoa frequently seen in the lumina of these ductules were totally negative for the various junctional proteins examined (e.g., Figs. 2, 3).

#### Seminiferous tubule epithelium

**Fig. 4:**
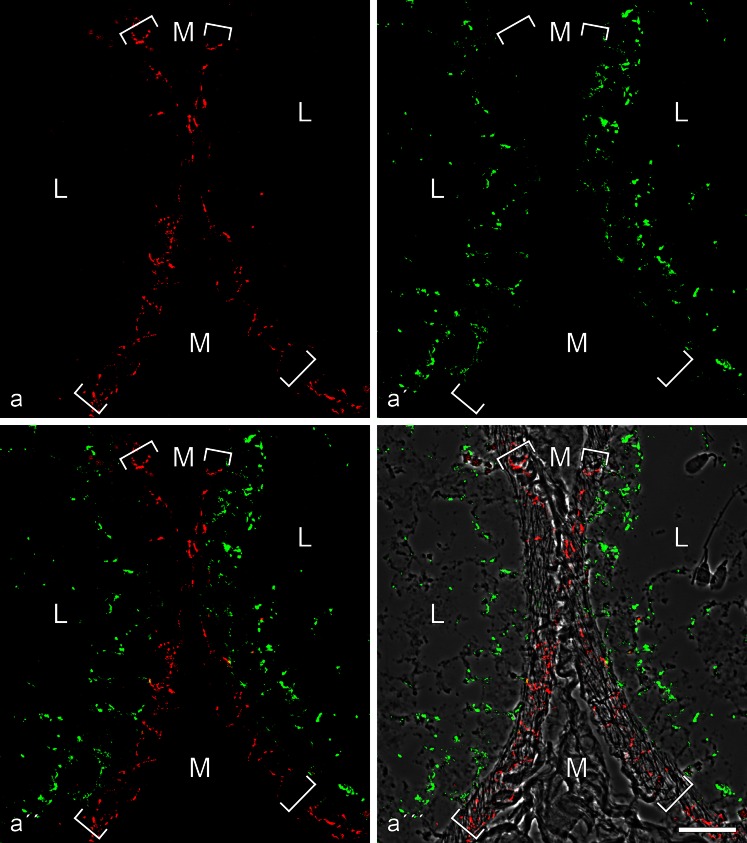
Double-label immunofluorescence microscopy of cryostat cross-sections through *tubuli seminiferi* of frozen bull testis after reactions with antibodies against E-cadherin (*red*; **a**, **a''**, **a'''**) and N-cadherin (*green*; **a'**, **a''**, **a'''**). In **a'''**, the reactions are shown on a phase contrast background. Note the mutually exclusive immunostaining of cell-cell junctions of the adherens type, N-cadherin-based ones (*green*) in the Sertoli cells and spermatogonia of the *tubuli seminiferi* and E-cadherin-containing junctions exclusively in a special layer of myoid cells surrounding the *tubuli* (demarcated by the *parentheses*). *M* mesenchymal region with interstitial cells; *L* lumen of the seminiferous tubules, with individual spermatids (e.g., on the *right-hand side* of **a'''**). *Bar* 20 μm

**Fig. 5:**
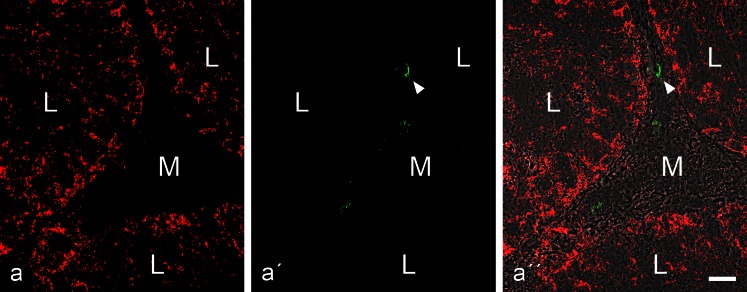
Double-label immunofluorescence microscopy showing the reactions of antibodies to N-cadherin (**a**, *red*, mouse mAb) and desmoplakin (**b**, *green*, guinea pig antibodies) on a cross-section through *tubuli seminiferi* of bull testis (*L*, tubular lumen; *M*, mesenchymal space). While the N-cadherin reaction identifies the AJs of the Sertoli and spermatogonial cell layer (**a** and **a''** show the reaction in the three neighbouring tubular structures) there is no desmoplakin reaction (**a'**; for a visualization “control” this picture has been selected as, accidentally, a very small artifical green particle is seen here, denoted by a *white* *arrowhead*). *Bar* 20 μm

**Fig. 6:**
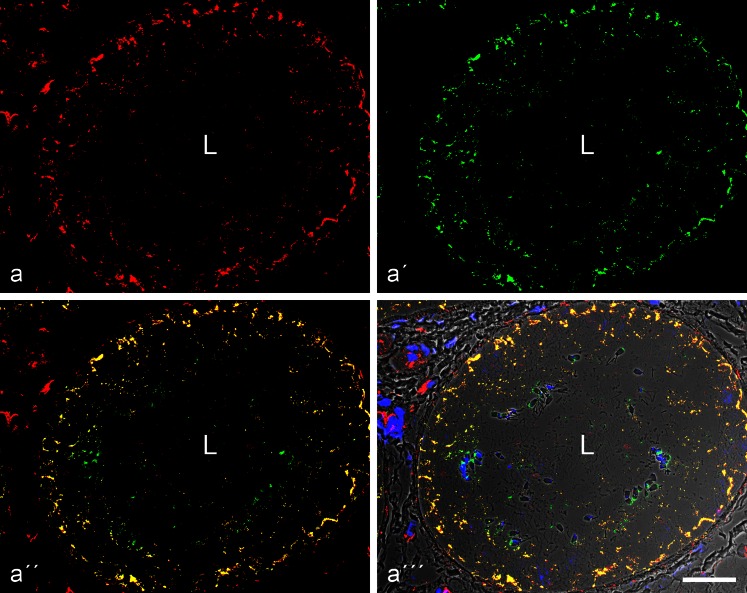
Double-label immunofluorescence microscopy of cross-sections through seminiferous tubules of frozen bull testis, showing the near-complete colocalization of β-catenin (**a**, *red*, mouse mAb) and N-cadherin (**a'**, *green*, rabbit antibodies) in the AJs of the Sertoli cell layer of the tubules, demonstrated by the *yellow* merged colour (**a''**, **a'''**; the latter is presented on a phase contrast background and with nuclei stained blue with DAPI). Note also β-catenin-positive structures in several types of interstitial cells. *L*, lumen. *Bar* 50 μm

**Fig. 7:**
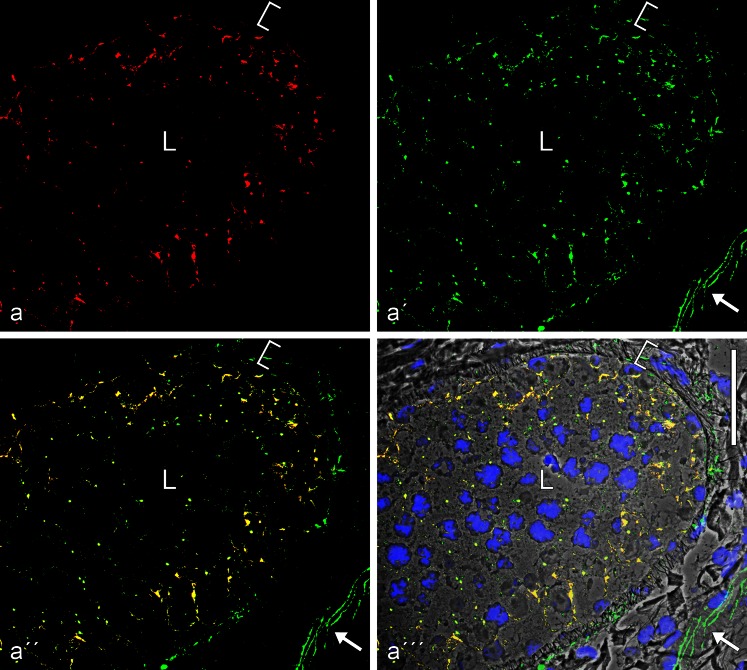
Double-label immunofluorescence microscopy of cross-sections through seminiferous tubules of frozen bull testis, showing the near-complete colocalization of N-cadherin (**a**, *red*, mouse mAb) and the plaque protein p0071 (**a'**, *green*, guinea pig antibodies) in the AJs of the Sertoli cells, demonstrated by the *yellow* merger colour (**a''**, **a'''**; the latter is shown on a phase contrast background and with DAPI-staining of nuclei). Note also the intensive green immunostaining of the p0071 reaction in the junctions connecting endothelial cells in blood and lymph vessels (here indicated, e.g., by the *arrow* in the *lower right-hand corner* of **a'**–**a'''**). *L*, lumen. *Bar* 50 μm

In correspondence with the biochemical results (Fig. 1), the tubule cells of the layer lining the adluminal side of the basal lamina were positive for both vimentin IFs and N-cadherin AJs (Electronic Supplementary Material, Figs. S2, S3), the latter in confirmation of Cyr et al. (1992, 1993), Newton et al. (1993) and Byers et al. (1994) but negative for keratins (for references see “Introduction”), E-cadherin and all the other cadherins examined (Figs. 4, 5, 6, and 7), including P-cadherin, VE-cadherin and cadherin-11 (Table 2; see also Cyr et al. 1992). In addition, a surprising positive E-cadherin reaction was detected in a thin non-epithelial cell layer of the interstitium of the bovine testis, a so-called “myoid” cell layer surrounding the tubules (red-stained cell-cell junctions in Fig. 4a–a'''), a layer also positive for keratins 8 and 18 (cf. Electronic Supplementary Material, Fig. S2).

**Fig. 8:**
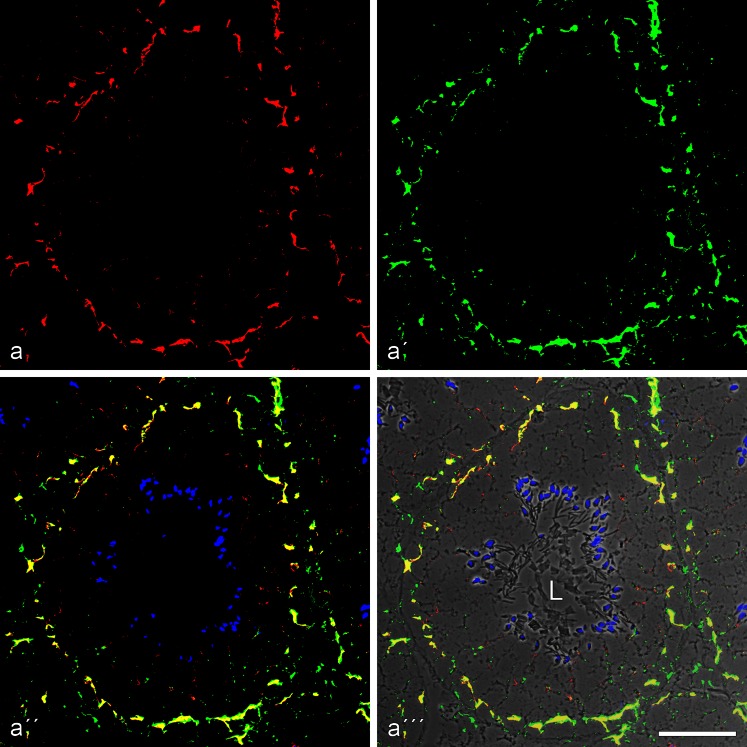
Double-label immunofluorescence microscopy of a cross-section through a seminiferous tubule of frozen mouse testis tissues, showing the very frequent colocalization of the plaque protein of the striatin family (**a**, *red*, mouse mAb) and β-catenin (**a'**, *green*, rabbit antibodies) in the basal parts of Sertoli cells and in spermatogonial cells, demonstrated by the yellow merger colour (**a''**, **a'''**; the latter is shown on a phase contrast background and with DAPI-staining of the nuclei). Note also the DAPI-stained elongated spermatid heads typical for rodents. *L*, lumen of the seminiferous tubules. *Bar* 50 μm

A very clear result of these immunolocalization experiments was that no significant reactions were seen for any of the cadherin family glycoproteins of either the desmoglein and the desmocollin group, in particular not for Dsg-2 and Dsc-2 (see, e.g., Electronic Supplementary Material, Fig. S4a). Also negative were the reactions for the desmosomal plaque proteins desmoplakin (Fig. 5) and plakophilin Pkp-2 (e.g., Electronic Supplementary Material, Fig. S4b), or any other member of the plakophilin group (Table 2). These negative results were obtained for all desmosomal marker molecules using antibodies that abundantly demonstrated positivity in all the species studied.

The N-cadherin-positive, punctate or elongated junction structures were positive for several of the cytoplasmic plaque proteins of the *armadillo* family such as β-catenin (Fig. 5), plakoglobin (not shown; see Table 2 and Byers et al. 1994), proteins p0071 (Figs. 6 and 7) and p120. They were also immunostained with antibodies to the actin-interacting protein α-catenin (for co-localization with β-catenin see Electronic Supplementary Material, Fig. S5; see also Table 2) as well as for the proteins myozap and striatin (Figs. 7 and 8; Table 2). On the other hand, the Sertoli cell membranes were totally negative for the transmembrane, junction-associated proteins PERP and EpCAM (Table 2; for comparison with positively stained cells, see, e.g., Rickelt et al. 2011; Pieperhoff et al. 2012; Franke et al. 2013).

### Electron microscopy

As some of the AJ-related structures in the seminiferous tubules are rather small, others very complex or extremely large and some of them also appear to be organized in cell-type-specific ways, they were examined in detail by transmission electron microscopy and immunoelectron microscopy. Only the AJ-type structures connecting Sertoli cells with each other or with spermatogenic cells will be dealt with in the present report.

**Fig. 9:**
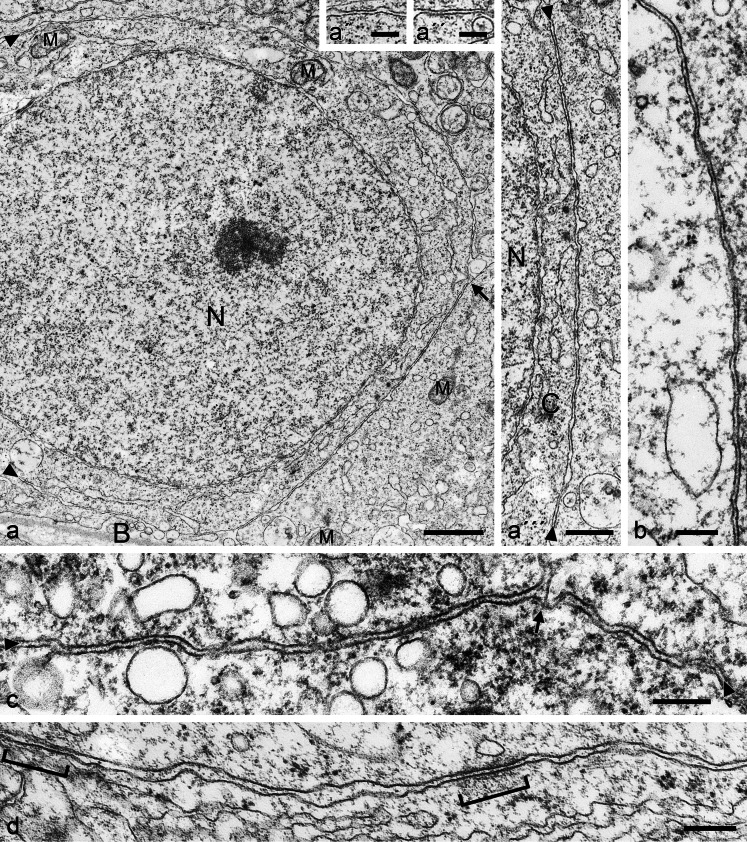
Electron micrographs of ultrathin sections through seminiferous tubules of boar (**a**–**a'''**) and bull (**b**–**d**) testis, showing a survey picture including large parts of a nucleus (*N*) and details of the very extended, rather regularly narrow-spaced (membrane-to-membrane interspace 8–18 nm) *area adhaerens* junctions; *B*, basal lamina; *M*, mitochondrion (**a'**–**a'''** present details at higher magnification; *C*, cytoplasm). Such extended, narrow-spaced plasma membrane connections of the “minimal plaque material” AJ type are also seen in bovine Sertoli cells (**b**–**d**) and only occasionally rather thin, loosely and irregularly arranged plaque-like structures are detected (see, e.g., **d**, *parentheses*). Note that these MPM-AJ associations are also maintained at sites where the plasma membranes of three cells meet (**c**, *arrow*). *Bars* (**a**) 1 μm, (**a'''**) 500 nm, (**a'**, **a''**, **b**–**d**) 200 nm

It is a striking observation that in well-fixed, optimally preserved *tubuli seminiferi*, the cells are intimately associated with each other by junction-like plasma membrane structures for very large proportions of the cell surface, often exceeding 50 % (e.g., Fig. 9a–a''', b). This rather consistently close and parallel contact with a membrane-to-membrane distance of 8–20 nm often appears to be accompanied by small and sparse submembranous densities, which are only loosely associated with the specific membrane regions (see, e.g., Fig. 9b–d). Such extended, close and parallel junctions are seen in both homotypic Sertoli cell associations as well as in Sertoli cell associations with spermatogenic cells.

**Fig. 10:**
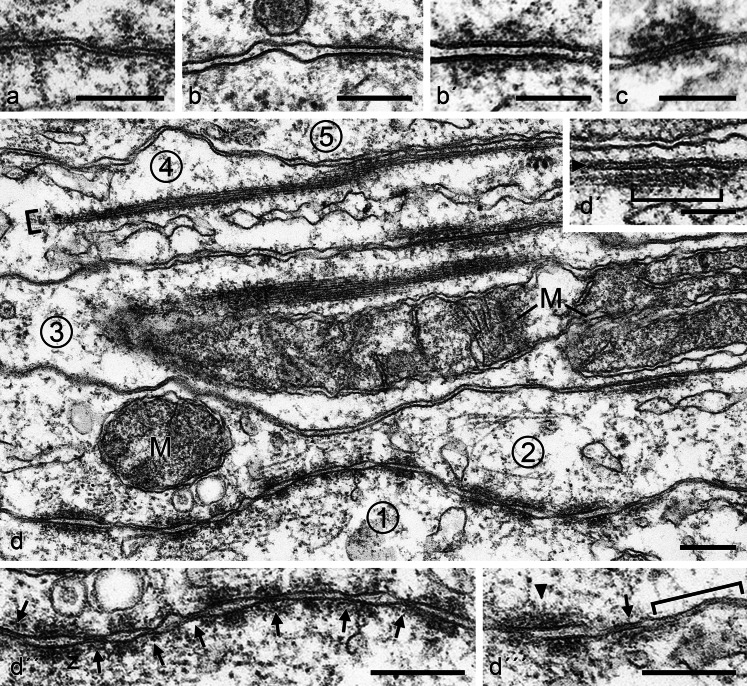
Electron micrographs of ultrathin sections through testicular tissue of a bull, showing various subtypes and aspects of AJs connecting Sertoli cells in the *tubuli seminiferi*. **a** Junctions characterized by a rather narrow distance between the membrane profiles (8–18 nm) loosely associated with some cytoplasmic plaque material that is highly variable in size and configuration. **b**, **b'** Overview (**b**) and partial magnification (**b'**) of a region containing an AJ with a strictly planar arrangement of 6- to 7-nm-thick membranes, an intermembrane space of 8–18 nm, with serially arranged “punctate midline” granules of 2–5 nm diameter (**b'**) and a general but loose cytoplasmic plaque coverage. **c** This small and rather narrow junction (ca. 5 nm intermembrane distance) is covered asymmetrically with irregularly shaped cytoplasmic plaque material. **d** Survey micrograph showing five tight-packed Sertoli cell processes and extended cell–cell contact regions (*areae adhaerentes*) between 5 pairs of cells (numbered *1*–*5*). Note in these Sertoli cell processes, the dense package of, e.g., mitochondria (*M*) and the so-called “ectoplasmic specializations”, i.e., cortical paracrystalline actin microfilament bundles that in some regions are parallel to—and rather closely associated with—cell–cell junction plasma membrane regions, often revealing lateral up to 4-nm-thick cross-bridges between the filament paracrystals and the plasma membrane (see, e.g., the bundle in the upper right of cell process numbered *2* and the *parenthesis* in the *insert* labeled **d'**). Note also the extended region with cell–cell junctions of the MPM-AJ type connecting cells numbered *1* and *2* in (**d**) (with higher partial magnifications in **d''** and **d'''**), showing numerous, closely spaced, dense arrangements of typical AJs with cytoplasmic plaque material separated by tight-adpressed special junctions of 10–30 nm diameter (*arrows* in **d''**, **d'''**). All three major junction types are seen side-by-side in (**d'''**): a *punctum adhaerens* (*arrowhead*), a tightly adpressed membrane junction (*arrow*) and a region of the MPM-AJ type (*parenthesis*; cf. Fig. 10). *Bars* (**a**, **b**, **d**, **d''**, **d'''**) 200 nm, (**b'**, **c**, **d'**) 100 nm

In some regions of such plaque-based cell–cell associations, local differences of the intermembranous space are notable (Figs. 9d, 10a–c) and, in some of these junction-like structures, a linear punctate midline array of granular-looking “dots” 2–5 nm in diameter is resolved (e.g., Fig. 10b, b', c; see also the junction denoted by an arrowhead in Fig. 10d'''). Closely spaced, small (diameters up to 500 nm) AJ-like structures with rather irregularly contoured, electron-dense plaques appear as almost regular arrays, interrupted only by some direct membrane-membrane “touch sites” of an as yet unidentified molecular nature (Fig. 10d, bottom: the series of AJ contacts between cell 1 and cell 2; for details, see also Fig. 10d'', d'''). Immunolocalization electron microscopy experiments published by Byers et al. (1991) and Pelletier and Byers (1992) have indicated the presence of protein ZO-1 at these sites that we can confirm. On the other hand, ZO-1–3 proteins cannot be considered unequivocal markers of tight junctions (TJs) as they have been identified in both TJ and AJ structures (for references. see, e.g., Franke 2010).

**Fig. 11:**
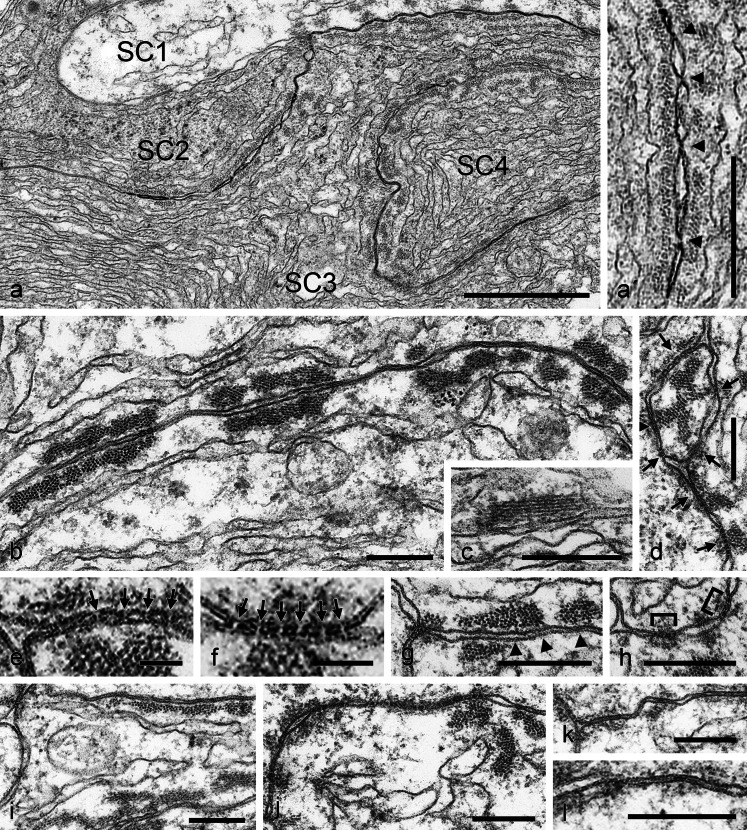
Electron micrographs of ultrathin sections through bovine testicular tissue, showing details of adherens regions (*areae adhaerentes*) and cribelliform junctions connecting Sertoli cells of a specific subtype. Sertoli cells of this subtype are characterized by a high packing density of endoplasmic reticulum cisternae in a cytoplasm of marked electron density and with extended regions of cell–cell junctions of the adherens type as well as some rather small cribelliform junctions and frequent junction-associated, paracrystalline actin microfilament bundles (“ectoplasmic specializations”). **a** Interdigitating processes of Sertoli cells (*SC1*–*SC4*) are connected by extended plasma membrane regions of “normal” intermembrane distance AJs, interspersed with small tightly adpressed membrane junctions some of which even suggest direct molecular interaction (**a'**, **b**, **d**). Distinct narrow channels between the cytoplasms of two Sertoli cells are indeed resolved in some very thin sections and appear as sieve-plate junctions (some positions are denoted by *arrows* in **d** and some of them are shown at higher magnification in **e** and **f**): cribelliform junctions (*areae cribelliformes*; **e**, **f**). The channel-like cell–cell continuities of these cribelliform junctions (**e**, **f**) have an inner “pore” diameter of 6–7 nm and a total length of 6–9 nm. Note that these cell–cell channels are often also characterized by electron-dense, plaque-like structures on one or on both sides of the channel (*arrowheads* or *brackets* in **d**, **f**, **h**, **j**). All in all, a major part of the plasma membrane indicates a junction-like association with adjacent actin filament bundles, which are often cross-bridged to the plasma membrane by short structures (**c**, **g**, **i**, **j**; see also *arrowheads* in **g**). Not infrequently, these parallel and close-spaced membrane-membrane junction-like structures are coated with loose and irregularly shaped cytoplasmic dense materials (**j**–**l**). *Bars* (**a**) 1 μm, (**a'**) 500 nm, (**b**–**d**, **g**–**l**) 200 nm, (**e**, **f**) 50 nm

Some of these close membrane-to-membrane associations (Fig. 10a–d show many such cell–cell contact regions between several Sertoli cell processes, numbered 1–5) are accompanied in a conspicuous way by so-called “ectoplasmic specializations” (ES; for morphological and immunocytochemical references, see, e.g., Dym and Fawcett 1970; Russell 1977c; Franke et al. 1978; Russell and Peterson 1985; Vogl 1989; Vogl et al. 1991, 2000, 2008; Mruk and Cheng 2004a; Wong et al. 2005; Yan et al. 2007; Kopera et al. 2010; Cheng and Mruk 2012; Qian et al. 2013). These are paracrystalline actin microfilament bundles that, in some regions, are directly connected to the inner side of the plasma membrane by lateral, rather closely spaced, up to 4-nm-thick cross-bridge structures (see, e.g., the inset in Fig. 10d'), whereas other portions of such bundles may protrude into the cytoplasm (see, e.g., the cells labelled nos. 3 and 4 in Fig. 10d). Microfilament bundles of such ES structures, associated with three or even more cells, were also repeatedly noted (e.g., Fig. 11d). Careful studies of the distributions of such ES structures have not allowed the identification of terminal associations (“anchorages”) at AJ plaque structures.

While we have not yet been able to elucidate the molecular composition of the “close contact” intercepts between the plaque-bearing, ES-associated AJ structures (cf., e.g., arrows and arrowheads in Figs. 10d'', d''', 11a), we often noted a fuzzy coating of electron-densely stained material at these structures (Figs. 10d, d'', d''', 11h–j). Obviously, these rather loose but regular associations with the “close” junctions have also impressed some previous authors who have even included them in model drawings of such structures (see, e.g., Pelletier and Byers 1992). A most surprising substructure in these cell–cell contact regions were the relatively small, sieve-plate-like cribelliform junctions (*areae cribelliformes*) characterized by regularly sized, membrane-bounded cell–cell “channels” or “pores”, often associated with short filamentous bundles (Fig. 11d–f; for similar short “bushes” of filaments, see also Fig. 3 of Connell 1978).

**Fig. 12:**
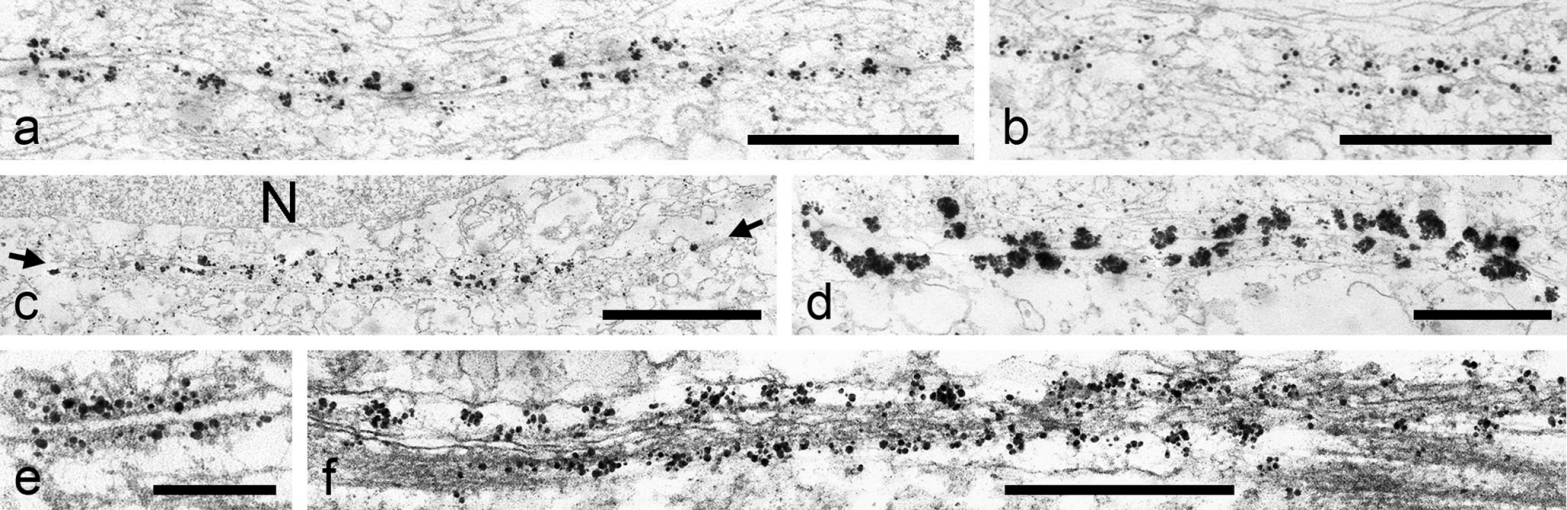
Immunoelectron microscopy of bull testicular tissue using antibodies against β-catenin (**a**–**d**), N-cadherin (**e**) and striatin (**f**). The silver-enhanced immunogold grains show specific binding of β-catenin in the extended regions of these Sertoli cell contacts with neighboring cells, including very long (4–6 μm) stretches with almost continuous β-catenin labelling (**a–d**). The reaction antibodies used in the preparation (**d**) have been enlarged by an especially intensive silver enhancement. All the diverse morphological subforms of Sertoli–Sertoli cell junctions also appear positive for N-cadherin (e.g., **e**) as well as for the other major adherens plaque proteins such as striatin (**f**) *Bars* (**c**) 1 μm, (**d**, **f**) 500 nm, (**a**, **b**, **e**) 200 nm

What was also conspicuous in these Sertoli cells was the abundance of bundles of vimentin IFs that did not insert at the AJs but were more or less parallel with respect to the plasma membrane as well as with the nuclear envelope (a typical overview is presented in Electronic Supplementary Material, Fig. S6; see also fig. 1 of Franke et al. 1979). Not infrequently, such vimentin IF bundles assumed almost paracrystalline order in which the IFs were separated by mean center-to-center distances of 10–25 nm (see, e.g., Electronic Supplementary Material, the bottom part of Fig. S6).

### Immunoelectron microscopy

Our immunoelectron microscopic results confirmed the negative immunofluorescence reactions of all desmosome-specific proteins and glycoproteins (desmoplakin, plakophilins, desmogleins, and desmocollins) in the adult *tubuli seminiferi* of all the species examined (not shown). By contrast, we saw more or less continuous immunogold labeling along the Sertoli cell associations with other Sertoli cells or with spermatogonial cells. Examples for the *armadillo* plaque protein β-catenin are shown in Fig. 12a–d (the continuous-appearing labeled region in Fig. 12a, for example, is more than 6 μm long; the regions shown in Fig. 12b–d are also very long). Similar results were obtained for N-cadherin (Fig. 12e) and the member of the striatin family present in the testis (Fig. 12f).

## Discussion

The Sertoli cells of the mature mammalian testis have to be classified as “epithelial” cells as they are based on a remarkably thick basal lamina, show a polar—basolateral-apical—architecture with lateral cell–cell junctions and border on a luminal space. On the other hand, during development and maturation, they have changed their molecular character as they have lost all keratin IFs and possess abundant bundles of vimentin IFs. They have also lost the typical epithelial junction architecture and instead are connected to each other and to the interspersed spermatogenic cells by a wealth of special and rather extended forms of adherens junctions.

### Absence of desmosomes and desmosome-specific molecules

One of the clearest and most important results of our study is the demonstration that the mature *tubuli seminiferi* of all five mammalian species examined do not contain any desmosome structures (*maculae adhaerentes* in the morphological definition of Farquhar and Palade 1963). Neither do they possess any of the desmosome-specific cadherins, i.e., desmogleins and desmocollins, nor any of the desmosome-specific cytoplasmic plaque proteins, i.e., desmoplakin or one of the plakophilins Pkp1-3. In contrast, plakoglobin as a protein component of plaques of both desmosomes and adherens junctions (AJs; cf., e.g., Cowin et al. 1986; Franke et al. 1987, 2009) is also found in AJs of Sertoli cells. So, in Sertoli and spermatogonial cells, there are no desmosome-like structures or related junctions with desmosome-typical molecules.

The significance of the absence of desmosomes and even of any desmosome-specific molecules in junctions connecting Sertoli cells with each other or with spermatogenic cells is also based on direct experimental comparisons with their abundant presence in all portions of the excurrent duct epithelia (see also, e.g., Table 2). So we hope that, from now on, words such as “desmosomes”, “desmosome-like structures” or “desmosomal proteins” will no longer appear in the literature on mature Sertoli cells (the need for this sentence is evident from Table 1 and Electronic Supplementary Material, Table S1), although the similar conclusion of Pelletier and Byers in 1992 (“Therefore, the term *desmosome-like* is seemingly inappropriate to designate these junctions…”) has already been widely ignored by researchers in this field.

Of course, we are aware of the fundamental controversy of our present report and the recent articles of several other authors (see Table 1 and Electronic Supplementary Material, Table S1), in particular the publication of Lie et al. (2010). These authors have specifically claimed, based on experiments at the mRNA (RT-PCR) and protein or glycoprotein (immunoblots and immunolocalization) level, that Sertoli cells of rats contain desmogleins Dsg-1, Dsg-2 and Dsg-4, desmocollins Dsc-1 and Dsc-3 and plakophilins Pkp-1, Pkp-2, and Pkp-4, as well as desmoplakin. While the mentioning of “plakophilin-4” as a desmosomal component is obsolete since it has been demonstrated that this plaque protein does not occur in any kind of desmosome but, as “protein p0071”, is restricted to AJs (Hofmann et al. 2008, 2009). In addition, the reported discovery of desmoglein Dsg-4 is highly disturbing as this glycoprotein has so far been identified only in the uppermost layers of the epidermis and in certain hair follicle layers (for references, see, e.g., Godsel et al. 2004; Schmidt and Koch 2008). However, as many of the claims reported by Lie et al. (2010) specifically address Sertoli cell cultures, a detailed critical discussion will have to be postponed to a future article in our series, which will deal with cell cultures and tumors assumed to be derived from Sertoli cells.

On the other hand, in this context, we do not forget to mention the specific occurrences of individual desmosomal molecules in other ensembles such as desmoplakin in the *complexus adhaerentes* of parts of lymphatic endothelia (Schmelz and Franke 1993; Hämmerling et al. 2006; Moll et al. 2009), plakophilin Pkp-2 in AJs of certain very proliferative stages of mesenchymal cells (e.g., Barth et al. 2009, 2012; Rickelt et al. 2010; Rickelt 2012) and the “free” Dsg-2 glycoproteins dispersed on the surfaces of certain types of melanoma cells (Schmitt et al. 2007; Rickelt et al. 2008). Particularly complex “hybrid junctions” are the composite junctions (*areae compositae*) connecting mammalian cardiomyocytes (Franke et al. 2006) and the “meningioma junctions” in which E- or N-cadherin can be associated not only with α- and β-catenin, plakoglobin and protein p120 but also with the “desmosomal protein”, plakophilin Pkp-2 (Akat et al. 2008).

The absence of desmosomes and any desmosome-specific molecules in mature Sertoli cells is also remarkable as these structures and molecules are present in stages of embryogenesis, fetal as well as early postnatal development, upon advanced aging and in various pathological situations and also in Sertoli-type cells of a wide variety of non-mammalian vertebrates (for references, see Baccetti et al. 1983; Bergmann et al. 1984; Pfeiffer and Vogl 2002).

Like keratin IF bundles, vimentin IF bundles can also be anchored at desmosomal plaques (e.g., Kartenbeck et al. 1984; Schwechheimer et al. 1984; Moll et al. 1986; for references, see Franke et al. 2009). As desmosomes are absent in mature Sertoli cells, it is thus noteworthy that the abundantly present vimentin IF bundles are not anchored at—nor in any other way firmly attached to—the plaques of the diverse subtypes of AJs aforementioned (see, e.g., Electronic Supplementary Material, Fig. S6). The same seems to be true for the neurofilament bundles (see also Davidoff et al. 1999).

### The various forms of AJs in the mammalian tubuli seminiferi, including the *areae adhaerentes*

Since the classic report by Farquhar and Palade (1963), the non-desmosomal adhering junctions, the adherens junctions (AJs), of epithelia have been found to occur in one of three morphological forms, the *zonula adhaerens*, the *fascia adhaerens* and the *punctum adhaerens*. Clearly, structures of the two latter types also exist in both the *tubuli seminiferi* and the excurrent duct epithelia, the excurrent ducts also having typical subapical *zonulae adhaerentes* (see also Figs. 1, 2, 3). In addition to these three forms, we now define a fourth major form, the *areae adhaerentes*, i.e., extended, often very large surface regions (e.g., including areas larger than 30 μm^2^ as determined from serial sections), which obviously provide very important structures of monolayer organization in the seminiferous tubules, both in homotypic AJs and in heterotypic connections with spermatogenic cells. As this study shows, they generally contain N-cadherin and the typical AJ plaque ensemble of proteins (Table 2). Whether such extended regions of the AJ type also occur in N-cadherin-based cell–cell contacts of other cells cannot yet be stated.

Morphologically, one can distinguish three major subforms of AJ structures in the seminiferous tubules (for diverse morphological AJ subtypes; see also fig. 28.1 of Pelletier 2001): One subform (type I) with a relatively thick and dense cytoplasmic plaque (e.g., Fig. 10d–d'''), a second subform (type II) with less and only loosely associated filamentous plaque substructures (e.g., Figs. 9d, 10a–c) and a third subform (type III) with very little and very thin cytoplasmic plaque filaments (MPM-AJs), which even in electron micrographs are often not distinctly resolved (e.g., Figs. 9, 10a–d, 11i–e). In certain areas of cell–cell contacts, diverse AJ subtypes may occur side by, side, often alternating or group-wise interspersed (e.g., Fig. 10d'''). These different AJ subforms also occur in the seminiferous tubules of many other mammalian species (see in particular Pelletier 1988, 2001; Pelletier and Byers 1992).

From a series of observations in the neuronal system, it may also be hypothesized that, in the seminiferous tubules, N-cadherin has in addition a developmental biological role in topogenesis and specific cell–cell interactions, including the local stabilization of other cell–cell connection structures, as it has first been proposed for certain synapses (e.g., Ushida et al. 1996; Arikkath 2010; Mendez et al. 2010; Tan et al. 2010; Gärtner et al. 2012a, b). Whether such roles and interactions can also be ascribed to some of the various subtypes of the N-cadherin–based junctional structures of the testis remains to be examined.

### Cribelliform junctions

With great surprise, we noticed, amidst the AJs of Sertoli cells, a further distinct and totally novel kind of cell–cell junction structure that is characterized by clusters of channel-like, ca. 6- to 7-nm-wide cytoplasmic continuity between adjacent Sertoli cells, i.e., pore structures formed by the fused plasma membranes of both cells. As we have shown, these sieve-plate-like, close-spaced assemblies of channels, *areae cribelliformes*, occur as small, distinct groups of cell–cell continuities that would allow the passage of relatively large molecules or particles. At present, we cannot answer questions as to their frequencies and functions or whether they contain any specific molecules, questions that we are currently trying to answer in quantitative and morphometric studies. Moreover, we will have to demonstrate the cell-to-cell passages of fluorescent or electron microscopic markers between Sertoli cells and determine the nature and sizes of molecules and particles that can take that route.

Of course, the question arises why these cribelliform junctions with their characteristic cytoplasmic sieve pore structures between Sertoli cells have not been described before. Here, however, careful examination of the published electron micrographs has revealed a few illustrations in works on guinea pig and dog seminiferous epithelia that show structures suggestive of such groups of cribelliform connections (see, e.g., figs. 3 and 4 of Connell 1978, or figs. 7 and 17 in Pelletier and Friend 1983). Thus, we expect that such junctions will be detectable in the Sertoli cells of diverse mammalian species.

### Concluding remarks

Our findings confirm and extend the view that the Sertoli cells of mature *tubuli seminiferi* and their cell–cell junction system represent a special and complex epithelial system, profoundly different from those of all other epithelial cells: Keratins are lost, desmosomal structures and desmosome-typical molecules are lost, E-cadherin is lost, EpCAM-containing junctions are lost. Instead, various subtypes of other *adhaerens* structures have formed that are based on N-cadherin. While such a transition from E- to N-cadherin is known from other cell-type changes in embryonal and fetal development as well as from certain pathological transformations as in the invasion and metastasis of diverse kinds of tumor cells (for review, see, e.g., Kalluri and Weinberg 2009), another form of advent of N-cadherin in epithelial cells, i.e., the formation of AJs based on E–N heterodimer clusters, as described for mammalian hepatocytes and liver tumor cells in situ and in culture (Straub et al. 2011), seems to be excluded from the mature and active Sertoli cells.

## Electronic supplementary material

Below is the link to the electronic supplementary material.

Fig. S1 Double-label immunofluorescence microscopy of an oblique cryostat cross-section through the upper region of an excurrent duct epithelium of a bull testis after reactions with antibodies against desmoglein 2, Dsg-2 (**a**, *red*, mouse mAb) and desmoplakin (**a'**, *green*, guinea pig pAb), demonstrating the sensitive and reliable desmosome identification by colocalization with both highly specific desmosome marker molecules, a transmembrane cadherin and a cytoplasmic plaque protein (**a''**, *yellow* merger colour). *L* lumen. *Bar* 20 μm (GIF 154 kb)

High resolution image (TIFF 1668 kb)

Fig. S2 Double-label immunofluorescence microscopy of seminiferous tubules in the testis of a sexually mature bull after cryofixation, followed by short treatment with acetone and PBS buffer containing 0.2% Triton X-100, 5 minute treatment with buffer containing 0.2% Triton X-100 and murine mAbs against vimentin, several washes, treatment with guinea pig antibodies to keratins 8 and 18, washes and incubation with the secondary antibodies for 5 minutes. All Sertoli cells (*L*, lumen; *M*, mesenchymal space) are intensely positive (*red*) for vimentin filament bundles but totally negative for any of the keratins (*green*). By contrast a rare type of keratin-positive cells of "myoid" cells in the interstitial mesenchymal region of the seminiferous tubules has resulted in a *yellow* merger colour by colocalization (see **a'-a'''**). *Bar* 20 μm (GIF 242 kb)

High resolution image (TIFF 2386 kb)

Fig. S3 Double-label immunofluorescence microscopy of cross-sections through seminiferous tubules of bovine (**a-a''**) and murine (**b, b'**) testis tissues (*L*, lumen; *M*, mesenchymal space), showing that both the N-cadherin-positive cell-cell junctions (*red*, mouse mAb) and the vimentin filament bundles (*green*, guinea pig antibodies) are positive in structures of the Sertoli cells that, however, overlap only in certain small regions (**a''**, *yellow* merger colour). Higher magnifications of the Sertoli cells (e.g., **b, b'**) show that the vimentin filaments extend over most of the cytoplasm (phase contrast background). *Bars* 20 μm (GIF 385 kb)

High resolution image (TIFF 4089 kb)

Fig. S4 Double-label immunofluorescence microscopy of a cross-section through a bull testis tissue (*L*, lumen of seminiferous tubules) after reactions with antibodies against desmoglein, Dsg-2 (**a**, *red*, murine mAb) and β-catenin (**a'**, *green*, rabbit antibodies) or plakophilin-2 (Pkp-2; **b**, *red*) and N-cadherin (**b**, rabbit antibodies). Note in the specific as well as the merged pictures (**a''**, **b''**) the complete absence of the desmosomal marker molecules, Dsg-2 and Pkp-2. *Bars* 20 μm (GIF 207 kb)

High resolution image (TIFF 2421 kb)

Fig. S5 Double-label immunofluorescence microscopy of cryostat sections through a seminiferous tubule of bull testis, presenting a demonstration of the colocalization of two adherens junction plaque proteins, α-catenin (**a**, murine mAb, *red*) and β-catenin (**a'**, rabbit antibodies, *green*). The colocalization is directly visible in the merged colour pictures (**a''**, **a'''** with phase contrast background). The nuclei in both the seminiferous tubule (*T*) and the interstitial cells of the surrounding mesenchyme have been stained *blue* with DAPI. *Bar* 20 μm (GIF 270 kb)

High resolution image (TIFF 3120 kb)

Fig. S6 Electron micrographs of ultrathin sections through Sertoli cells of bull testis, showing the nucleus (*N*) with nucleoplasm and chromatin, mitochondria (*M*), the dense cytoskeletal coverage of the nuclear envelope with bundles of vimentin filaments (*V* and *bracket* in the upper left), endoplasmic reticulum cisternae, an extended plasma membrane cell-cell contact region (*arrowheads*) with plaque-bearing adherens junction (AJ) structures (*three brackets* on the right hand edge; see also the insert, **b**) and very small AJ "midline" structures (*insert*). The vimentin filaments often can come near tocell-cell junctions but do not anchor at – or otherwise attach to – any AJ structures (**b**, *bracket* in the upper left denotes an intermediate filament bundle). *Bars* 500 nm (**a**), 200 nm (**b**) (GIF 310 kb)

High resolution image (TIFF 4573 kb)

Table S1 Reports claiming that desmosomes or desmosome-like junctions occur in the tubuli seminiferi of mammalian testes (only references since 1983 are considered here as identifications using molecule-specific antibodies against desmosomal components have been generally available since that year). (DOC 36 kb)

Table S2 Primary Antibodies mAb: monoclonal antibody; pAb: polyclonal antibodies; m: mouse; rb: rabbit; gp: guinea pig. (DOC 185 kb)
