# Supplementary figures and images for: The cell–cell junctions of mammalian testes: I. The adhering junctions of the seminiferous epithelium represent special differentiation structures
Source: Cell Tissue Res. 2014 Jun 8;357(3):645–65. doi: 10.1007/s00441-014-1906-9 (PMC4148596; doi:10.1007/s00441-014-1906-9)

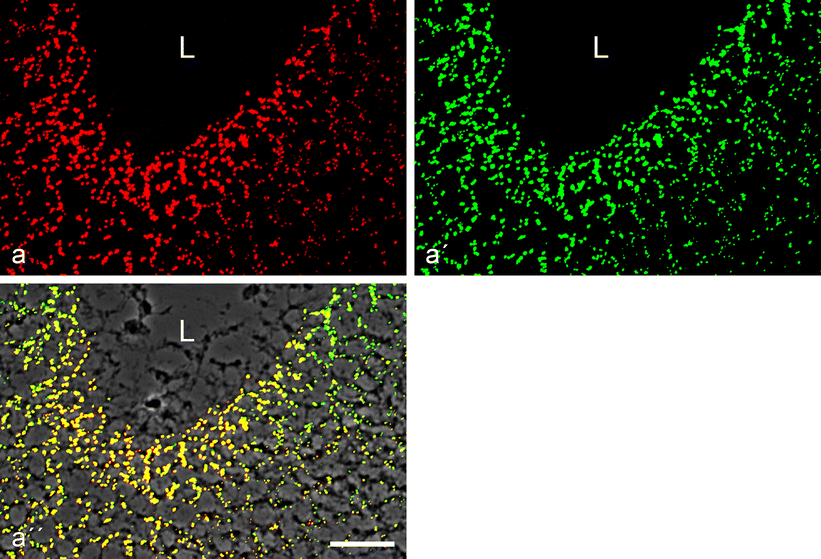

Supplement: Supplementary file 1 — Double-label immunofluorescence microscopy of an oblique cryostat cross-section through the upper region of an excurrent duct epithelium of a bull testis after reactions with antibodies against desmoglein 2, Dsg-2 (a, red, mouse mAb) and desmoplakin (a', green, guinea pig pAb), demonstrating the sensitive and reliable desmosome identification by colocalization with both highly specific desmosome marker molecules, a transmembrane cadherin and a cytoplasmic plaque protein (a'', yellow merger colour). L lumen. Bar 20 μm (GIF 154 kb) [file 441_2014_1906_Fig13_ESM.gif]

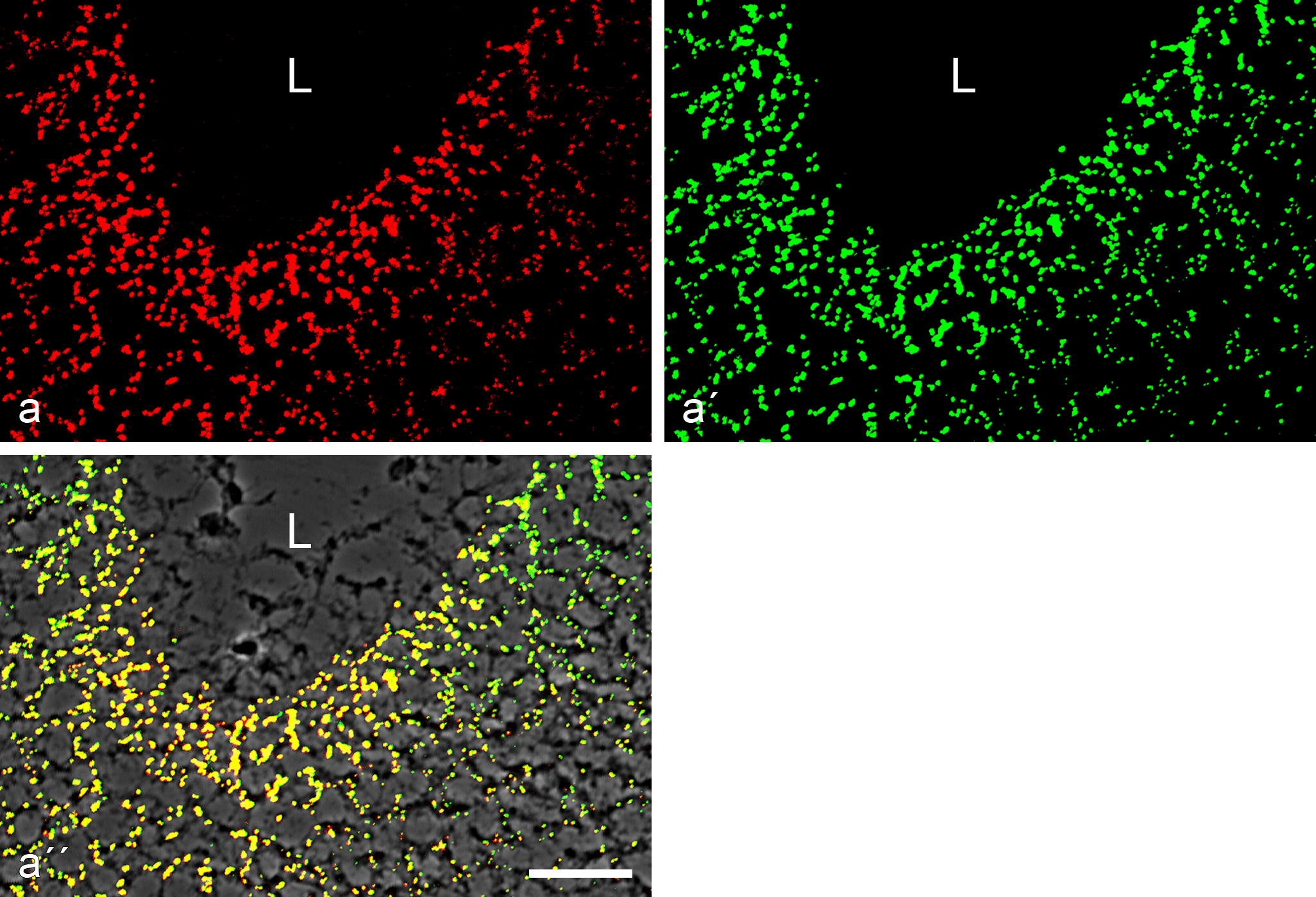

Supplement: Supplementary file 2 — High resolution image (TIFF 1668 kb) [file 441_2014_1906_MOESM1_ESM.tif]

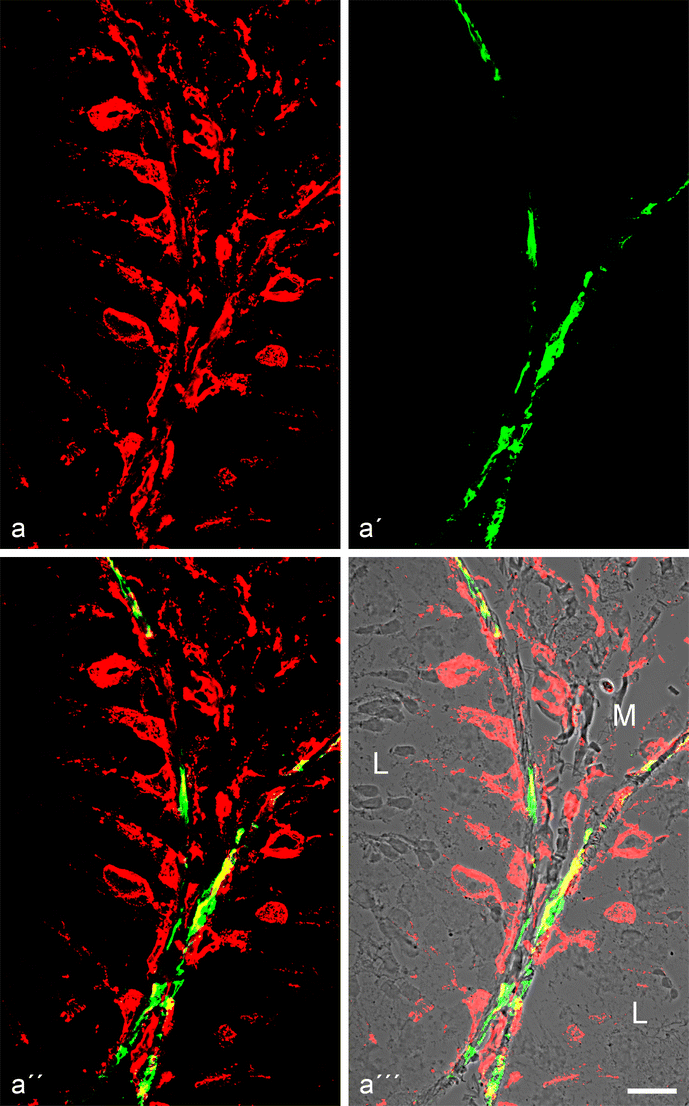

Supplement: Supplementary file 3 — Double-label immunofluorescence microscopy of seminiferous tubules in the testis of a sexually mature bull after cryofixation, followed by short treatment with acetone and PBS buffer containing 0.2% Triton X-100, 5 minute treatment with buffer containing 0.2% Triton X-100 and murine mAbs against vimentin, several washes, treatment with guinea pig antibodies to keratins 8 and 18, washes and incubation with the secondary antibodies for 5 minutes. All Sertoli cells (L, lumen; M, mesenchymal space) are intensely positive (red) for vimentin filament bundles but totally negative for any of the keratins (green). By contrast a rare type of keratin-positive cells of "myoid" cells in the interstitial mesenchymal region of the seminiferous tubules has resulted in a yellow merger colour by colocalization (see a'-a'''). Bar 20 μm (GIF 242 kb) [file 441_2014_1906_Fig14_ESM.gif]

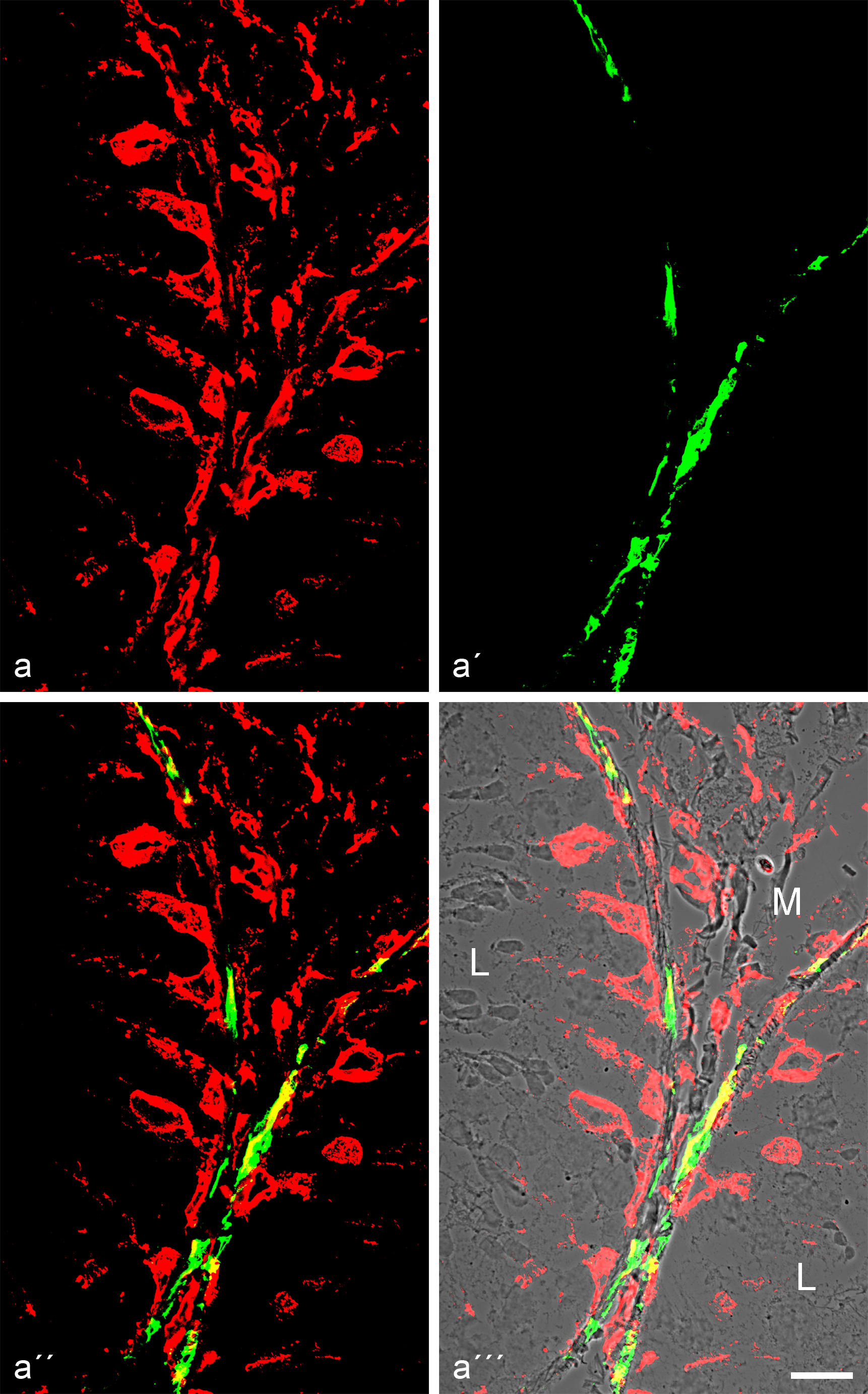

Supplement: Supplementary file 4 — High resolution image (TIFF 2386 kb) [file 441_2014_1906_MOESM2_ESM.tif]

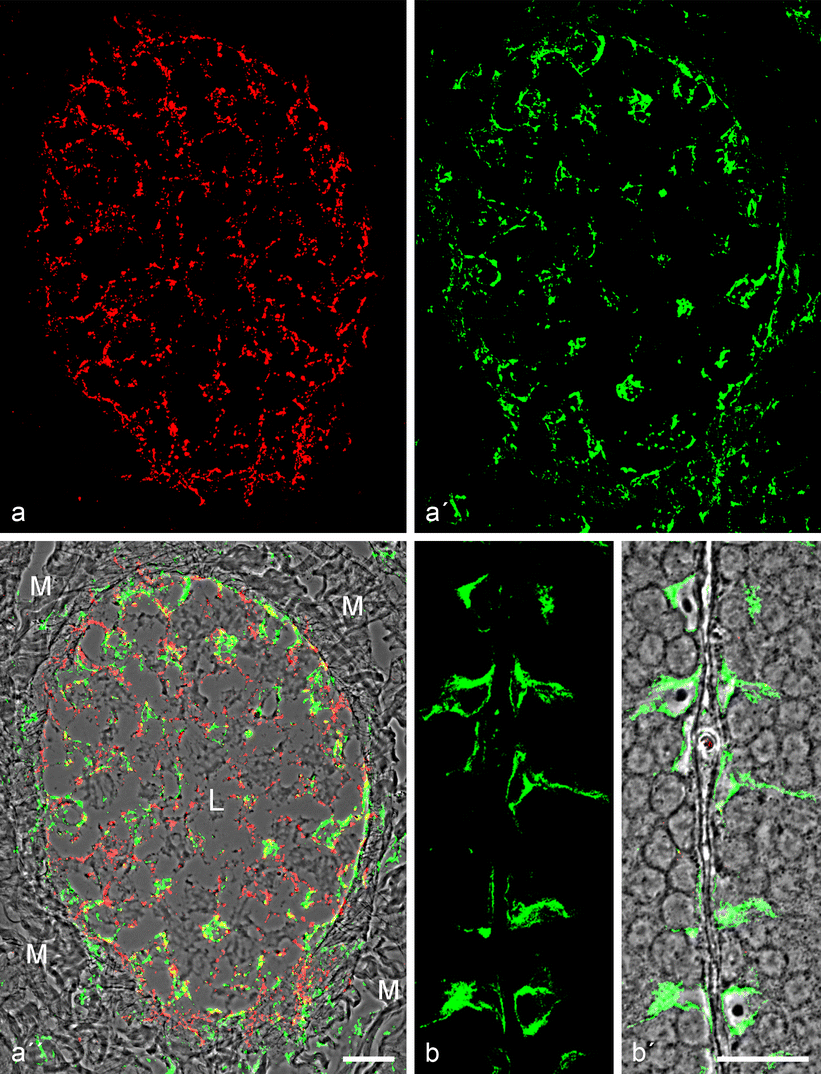

Supplement: Supplementary file 5 — Double-label immunofluorescence microscopy of cross-sections through seminiferous tubules of bovine (a-a'') and murine (b, b') testis tissues (L, lumen; M, mesenchymal space), showing that both the N-cadherin-positive cell-cell junctions (red, mouse mAb) and the vimentin filament bundles (green, guinea pig antibodies) are positive in structures of the Sertoli cells that, however, overlap only in certain small regions (a'', yellow merger colour). Higher magnifications of the Sertoli cells (e.g., b, b') show that the vimentin filaments extend over most of the cytoplasm (phase contrast background). Bars 20 μm (GIF 385 kb) [file 441_2014_1906_Fig15_ESM.gif]

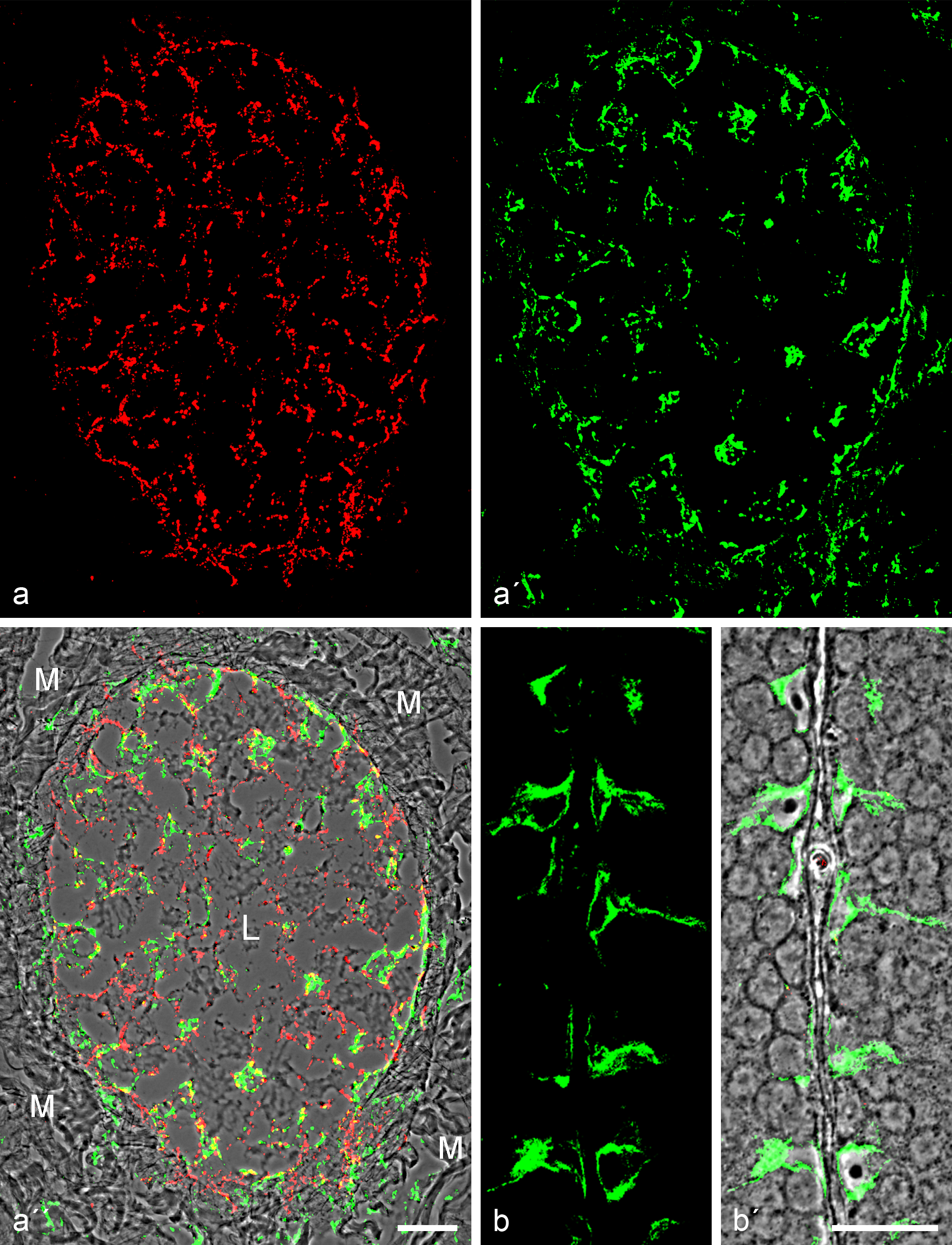

Supplement: Supplementary file 6 — High resolution image (TIFF 4089 kb) [file 441_2014_1906_MOESM3_ESM.tif]

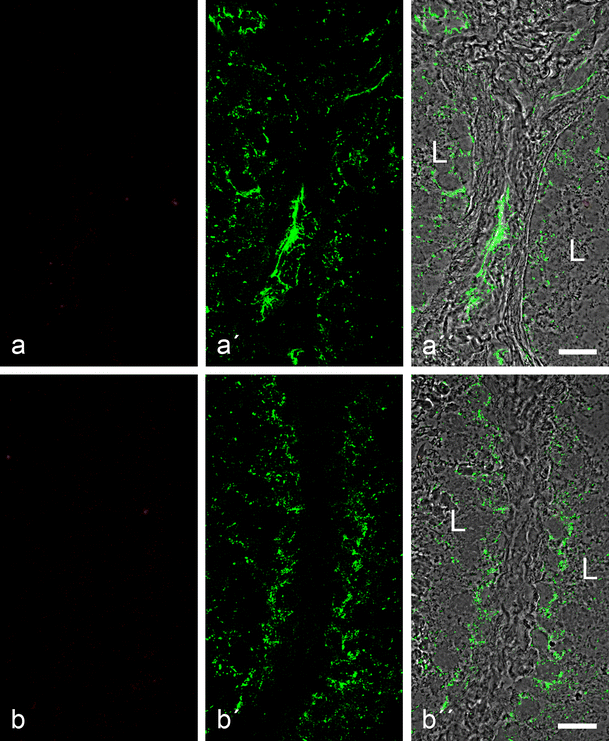

Supplement: Supplementary file 7 — Double-label immunofluorescence microscopy of a cross-section through a bull testis tissue (L, lumen of seminiferous tubules) after reactions with antibodies against desmoglein, Dsg-2 (a, red, murine mAb) and β-catenin (a', green, rabbit antibodies) or plakophilin-2 (Pkp-2; b, red) and N-cadherin (b, rabbit antibodies). Note in the specific as well as the merged pictures (a'', b'') the complete absence of the desmosomal marker molecules, Dsg-2 and Pkp-2. Bars 20 μm (GIF 207 kb) [file 441_2014_1906_Fig16_ESM.gif]

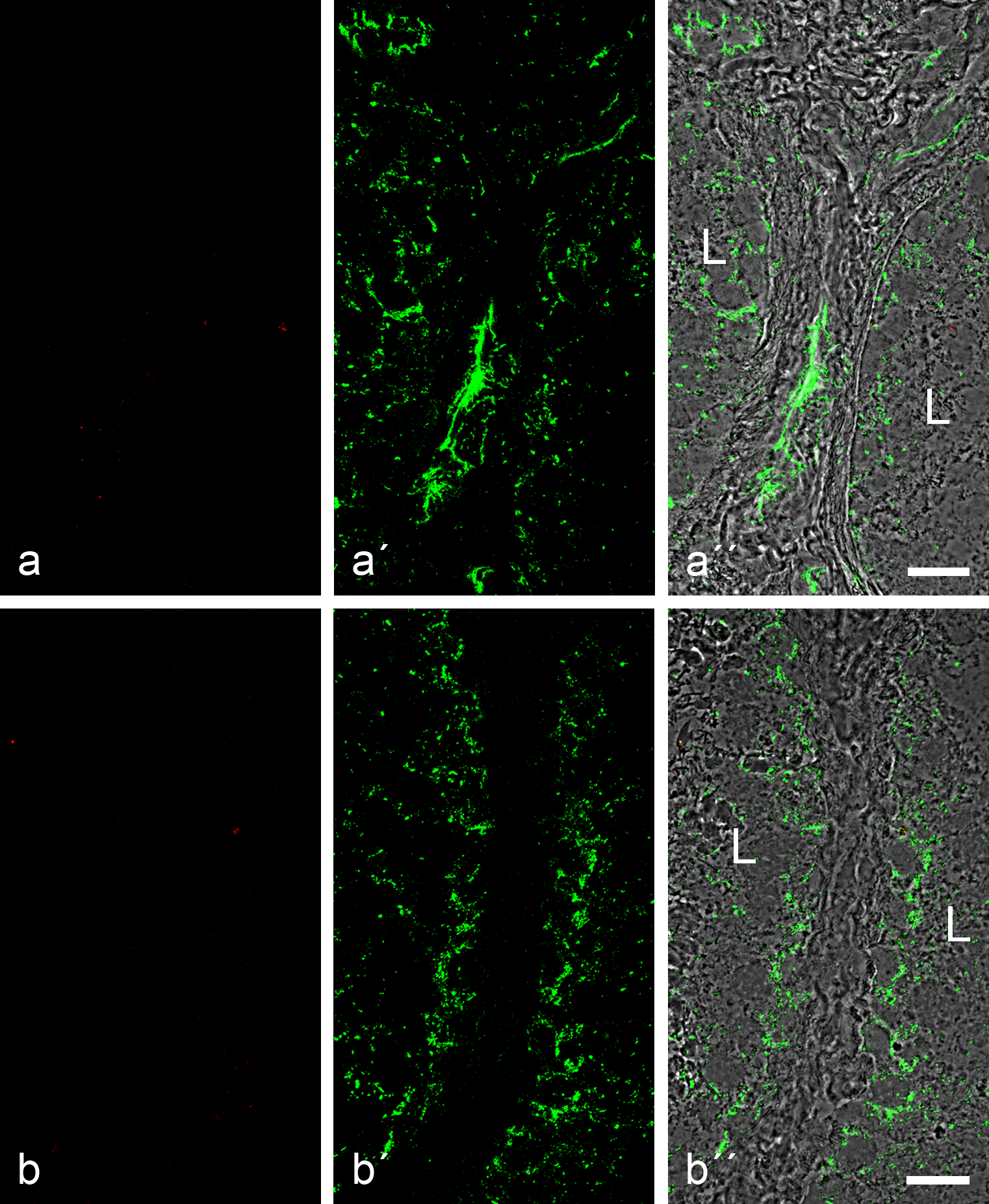

Supplement: Supplementary file 8 — High resolution image (TIFF 2421 kb) [file 441_2014_1906_MOESM4_ESM.tif]

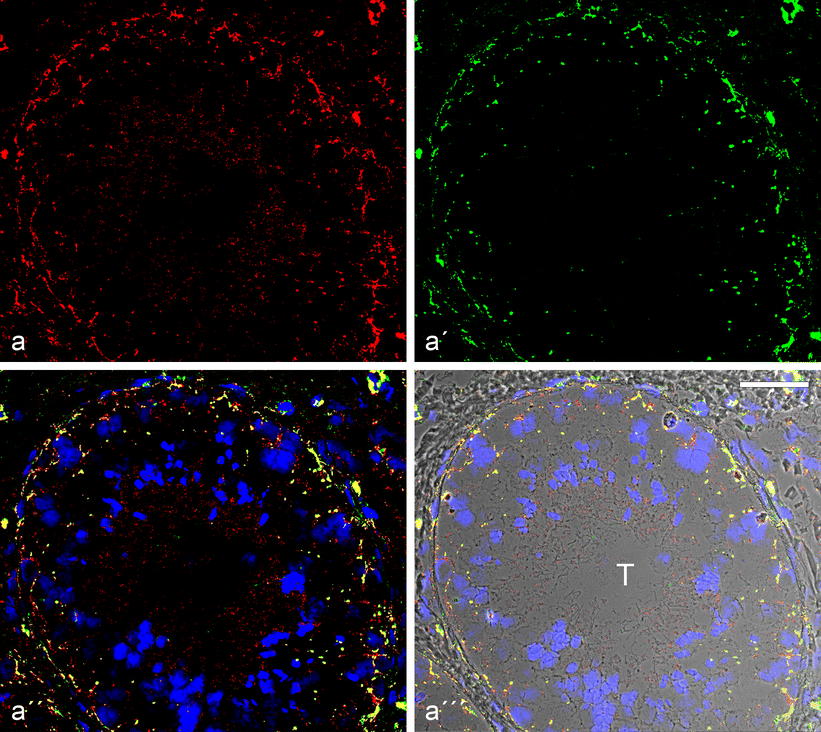

Supplement: Supplementary file 9 — Double-label immunofluorescence microscopy of cryostat sections through a seminiferous tubule of bull testis, presenting a demonstration of the colocalization of two adherens junction plaque proteins, α-catenin (a, murine mAb, red) and β-catenin (a', rabbit antibodies, green). The colocalization is directly visible in the merged colour pictures (a'', a''' with phase contrast background). The nuclei in both the seminiferous tubule (T) and the interstitial cells of the surrounding mesenchyme have been stained blue with DAPI. Bar 20 μm (GIF 270 kb) [file 441_2014_1906_Fig17_ESM.gif]

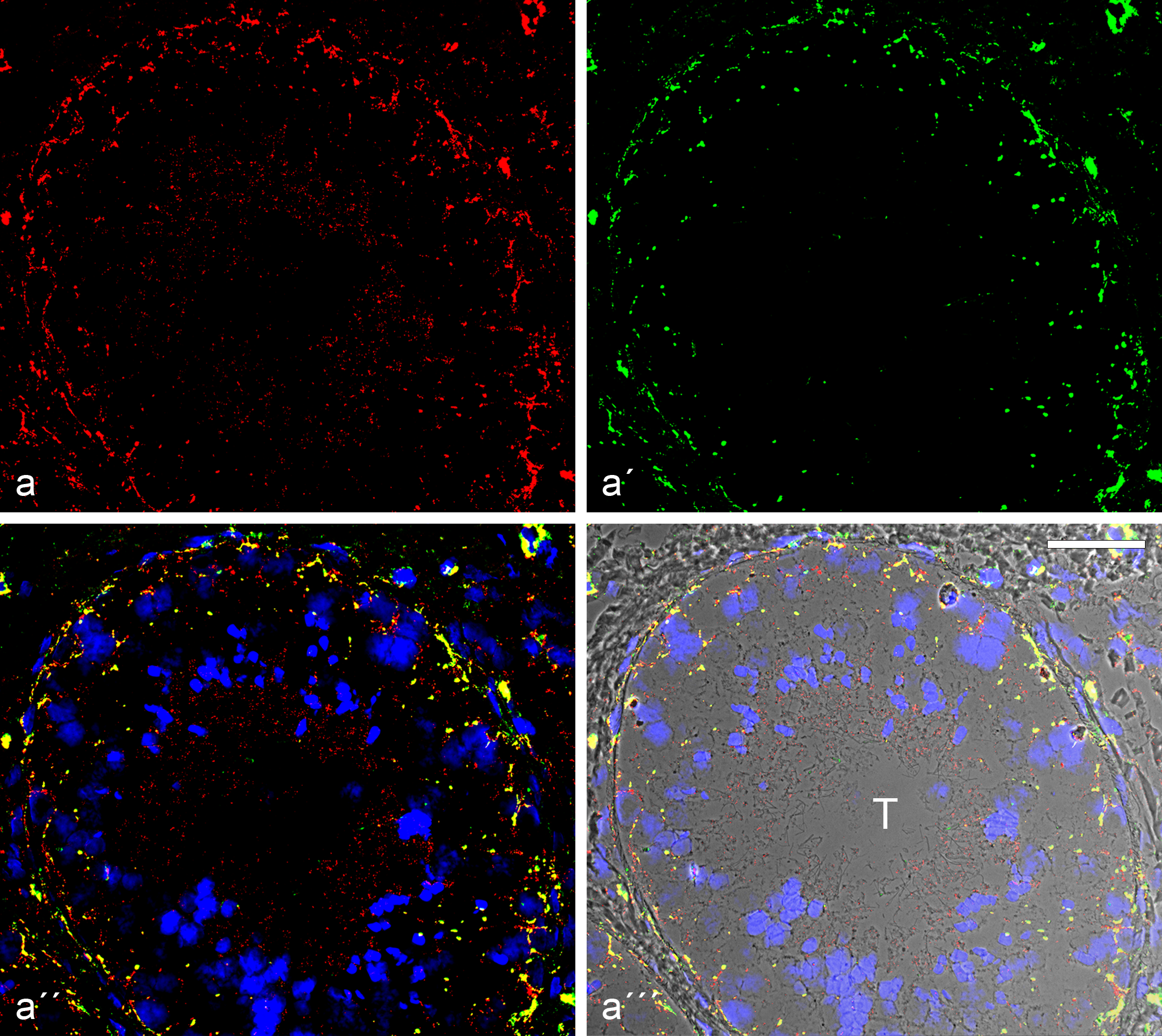

Supplement: Supplementary file 10 — High resolution image (TIFF 3120 kb) [file 441_2014_1906_MOESM5_ESM.tif]

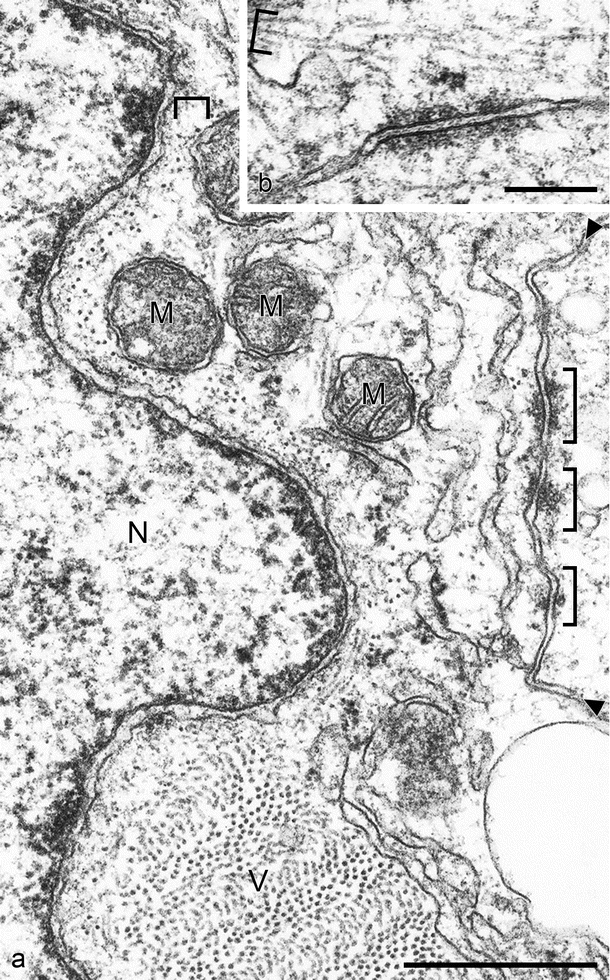

Supplement: Supplementary file 11 — Electron micrographs of ultrathin sections through Sertoli cells of bull testis, showing the nucleus (N) with nucleoplasm and chromatin, mitochondria (M), the dense cytoskeletal coverage of the nuclear envelope with bundles of vimentin filaments (V and bracket in the upper left), endoplasmic reticulum cisternae, an extended plasma membrane cell-cell contact region (arrowheads) with plaque-bearing adherens junction (AJ) structures (three brackets on the right hand edge; see also the insert, b) and very small AJ "midline" structures (insert). The vimentin filaments often can come near tocell-cell junctions but do not anchor at – or otherwise attach to – any AJ structures (b, bracket in the upper left denotes an intermediate filament bundle). Bars 500 nm (a), 200 nm (b) (GIF 310 kb) [file 441_2014_1906_Fig18_ESM.gif]

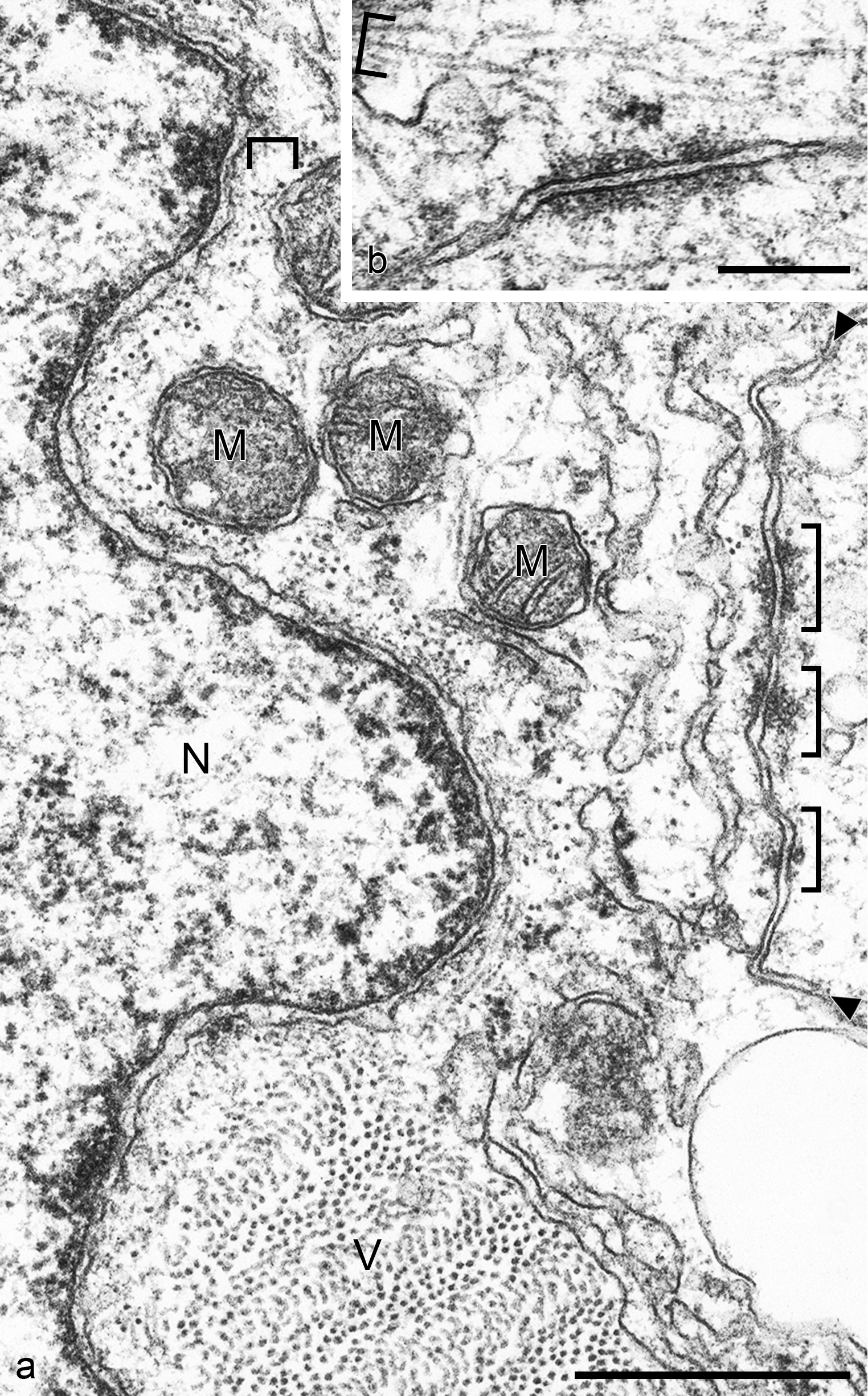

Supplement: Supplementary file 12 — High resolution image (TIFF 4573 kb) [file 441_2014_1906_MOESM6_ESM.tif]
