# Supplementary material for: The cell–cell junctions of mammalian testes: I. The adhering junctions of the seminiferous epithelium represent special differentiation structures
Source: Cell Tissue Res. 2014 Jun 8;357(3):645–65. doi: 10.1007/s00441-014-1906-9 (PMC4148596; doi:10.1007/s00441-014-1906-9)
Supplement: Supplementary file 13 — Reports claiming that desmosomes or desmosome-like junctions occur in the tubuli seminiferi of mammalian testes (only references since 1983 are considered here as identifications using molecule-specific antibodies against desmosomal components have been generally available since that year). (DOC 36 kb) [file 441_2014_1906_MOESM7_ESM.doc]

**Supplementary Table S1**

**Reports claiming that desmosomes or desmosome-like junctions occur in the *tubuli seminiferi* of mammalian testes (only references since 1983 are considered here as identifications using molecule-specific antibodies against desmosomal components have been generally available since that year).**

**(Complete references for Table 1)**

Alves MG, Martins AD, Cavaco JE, Socorro S, Oliveira (2013) Diabetes, insulin-mediated glucose metabolism and Sertoli/blood-testis barrier function. Tissue Barriers 1:e23992

Bergmann M, Schindelmeiser J, Greven H (1984) The blood-testis barrier in vertebrates having different testicular organization. Cell Tissue Res 238.145-150

Chapin RE, Wine RN, Harris MW, Borchers CH, Haseman JK (2001) Structure and control of a cell-cell adhesion complex associated with spermiation in rat seminiferous epithelium. J Androl 22:1030-1052

Cheng CY, Mruk DD (2002) Cell junction dynamcis in the testis: Sertoli-germ cell interactions and male contraceptive development. Physiol Rev 82:825-874

Cheng CY, Mruk DD (2011) Regulation of spermiogenesis, spermiation and blood-testis barrier dynamics: novel insights from studies on Eps8 and Arp3. Biochem J 435:553-562

Cheng CY, Mruk DD (2012) The blood-testis barrier and its implications for male contraception. Pharmacol Rev 64:16-64

Cheng CY, Wong EWP, Lie PPY, Li MWM Mruk DD, Yan HHN, Mok D-W, Mannu J, Mathur PP, Lui W-y, Bonanomi M, Silvestrini B (2011) Regulation of blood-testis barrier dynamics by desmosome, gap junction, hemidesmosome and polarity proteins. An unexpected turn of events. Spermatogenesis 1:105-115

Cheng CY, Lie PPY, Wong EWP, Mruk DD (2013) Focal adhesion kinase and actin regulatory/binding proteins that modulate F-actin organization at the tissue barrier. Tissue Barriers 1:e24252

Goossens S, van Roy F (2005) Cadherin-mediated cell-cell adhesion in the testis. Front Biosci 10:398-419

Johnson KJ, Boekelheide K (2002a) Dynamic testicular adhesion junctions are immunologically unique. I. Localization of p120 catenin in rat testis. Biol Reprod 66:983-991

Johnson KJ, Boekelheide K (2002b) Dynamic testicular adhesion junctions are immunologically unique. II. Localization of classic cadherins in rat testis. Biol Reprod 66:992-1000

Kopera IA, Bilinska B, Cheng CY, Mruk DD (2010) Sertoli-germ cell junctions in the testis: a review of recent data. Phil Trans R Soc B 365:1593-1605

Lee NPY, Cheng CY (2004) Ectoplasmic specialization, a testis-specific cell-cell actin-based adherens junction type: is this a potential target for male contraceptive development? Hum Reprod Update 10:349-369

Lee NPY, Wong EWP, Mruk DD, Cheng CY (2009) Testicular cell junction: a novel target for male contraception. Curr Med Chem 16:906-915

Li JCH, Mruk D, Cheng CY (2001) The inter-Sertoli tight junction permeability barrier is regulated by the interplay of protein phosphatases and kinases: an in vitro study. J Androl 22:847-856

Li MWM, Mruk DD, Lee WM, Cheng CY (2009) Connexin 43 and plakophilin-2 as a protein complex that regulates blood-testis barrier dynamics. Proc Natl. Acad Sci USA 106:10213-10218

Li MWM, Mruk DD, Lee WM, Cheng CY (2010) Connexin 43 is critical to maintain the homeostasis of the blood-testis barrier via its effects on tight junction reassembly. Proc Natl Acad Sci USA 107:17998-18003

Lie PPY, Cheng CY, Mruk DD (2010) Crosstalk between desmoglein-2/desmocollin-2/Src kinase and coxsackie and adenovirus receptor/ZO-1 protein complexes regulates blood-testis barrier dynamics. Int J Biochem Cell Biol 42:975-986

Lie PPY, Cheng CY, Mruk DD (2011) The biology of the desmosome-like junction: a versatile anchoring junction and signal transducer in the seminiferous epithelium. Int Rev Cell Mol Biol 286:223-269

Mok K-W, Mruk DD, Lee WM, Cheng CY (2013) Rictor/mTORC2 regulates blood-testis barrier dynamics via its effects on gap junction communications and actin filament network. FASEB J 27:1137-1152

Morrow CMK, Mruk D, Cheng CY, Hess RA (2010) Claudin and occludin expression and function in the seminiferous epithelium. Phil Trans R Soc B 365:1679-1696

Mruk DD, Cheng CY (2004a) Cell-cell interactions at the ectoplasmic specialization in the testis. Trends Endocrinol Metab 15:439-447

Mruk DD, Cheng CY (2004b) Sertoli-Sertoli and Sertoli-germ cell interactions and their significance in germ cell movement in the seminiferous epithelium during spermatogenesis.Endocr Rev 25:747-806

Mruk DD Cheng CY (2011) Desmosomes in the testis. Moving into an unchartered territory. Spermatogenesis 1:47-51

Mruk DD, Xiao X, Lydka M, Li MWM, Bilinska B, Cheng CY (2013) Intercellular adhesion molecule 1: Recent findings and new concepts involved in mammalian spermatogenesis. Sem Cell Dev Biol, in press. Published online 10 August 2013, http://dx.doi.org/10.1016/j.semcdb.2013.07.003

Mullholland DJ, Dedhar S, Vogl AW (2001) Rat seminiferous epithelium contains a unique junction (ectoplasmic specialization) with signaling properties both of cell/cell and cell/matrix junctions. Biol Reprod 64:396-407

Osman DI (1978) On the ultrastructure of modified Sertoli cells in the terminal segment of seminiferous tubules in the boar. J Anat 127:603-613

Russell LD, Peterson RN (1985) Sertoli cell junctions: Morphological and functional correlates. Int Rev Cytol 94:177-211

Su W, Mruk DD, Cheng CY (2013) Regulation of actin dynamics and protein trafficking during spermatogenesis – insights into a complex process. Crit Rev Biochem Mol Biol 48:153-172

Vogl AW, Pfeiffer DC, Mulholland D, Kimel G, Guttman J (2000) Unique and multifunctional adhesion junctions in the testis: ectoplasmic specializations. Arch Histol Cytol 63:1-15

Vogl AW, Vaid KS, Guttman JA (2008) The Sertoli cell cytoskeleton. Adv Exp Med Biol 636:186-211

Vogl AW, Du M, Wang E, Young JS (2013a) Novel clathrin/actin-based endocytic machinery associated with junction turnover in the seminiferous epithelium. Sem Cell Dev Biol. In press, http://dx.doi.org/10.1016/j.semcdb.2013.11.002

Vogl AW, Young JS, Du M (2013b) New insights into roles of tubulobulbar complexes in sperm release and turnover of blood-testis barrier. Int Rev Cell Mol Biol 303:319-355

Wine RN, Chapin RE (1999) Adhesion and signaling proteins spatiotemporally associated with spermiation in the rat. J Androl 20:198-213

Wong C-H, Cheng CY (2005) The blood-testis barrier: its biology, regulation, and physiological role in spermatogenesis. Curr Top Dev Biol 71:263-296

Wong C-h, Mruk DD, Lui W-y, Cheng CY (2004) Regulation of blood-testis barrier dynamics: an in vivo study. J Cell Sci 117:783-798

Wong C-H, Xia W, Lee, NPY, Mruk DD, Lee MW, Cheng CY (2005) Regulation of ectoplasmic specialization dynamics in the seminiferous epithelium by focal adhesion-associated proteins in testosterone-suppressed rat testes. Endocrinology 146:1192-12014

Xia W, Wong CH, Lee NPY, Lee WM, Cheng CY (2005) Disruption of Sertoli-germ cell adhesion function in the seminiferous epithelium of the rat testis can be limited to adherens junctions without affecting the blood-testis barrier integrity: an in vivo study using an androgen suppression model. J Cell Physiol 205:141-157

# Yan HH, Cheng CY (2005) Blood-testis barrier dynamics are regulated by an engagement/disengagement mechanism between tight and adherens junctions via peripheral adaptors. Proc Natl Acad Sci USA 102:11722-11727

Yan HHN, Mruk DD, Lee WM, Cheng CY (2007) Ectoplasmic specializations: a friend or foe of spermatogenesis? BioEssays 29:36-48

Zhang J, Mruk DD, Cheng CY (2005a) Myotubularin phosphoinositide phosphatases, protein phosphatases, and kinases: their roles in junction dynamics and spermatogenesis. J Cell Physiol 204:470-483

Zhang J, Wong C-h, Xia W, Mruk DD, Lee NPY, Lee WM, Cheng CY (2005b) Regulation of Sertoli-germ cell adherens junction dynamics via changes in protein-protein interactions of the N-cadherin-β-catenin protein complex which are possibly mediated by c-Src and myotubularin-related protein 2: an in vivo study using an androgen suppression model. Endocrinology 146:1268-1284
