# Supplementary material for: The cell–cell junctions of mammalian testes: I. The adhering junctions of the seminiferous epithelium represent special differentiation structures
Source: Cell Tissue Res. 2014 Jun 8;357(3):645–65. doi: 10.1007/s00441-014-1906-9 (PMC4148596; doi:10.1007/s00441-014-1906-9)
Supplement: Supplementary file 14 — Primary Antibodies mAb: monoclonal antibody; pAb: polyclonal antibodies; m: mouse; rb: rabbit; gp: guinea pig. (DOC 185 kb) [file 441_2014_1906_MOESM8_ESM.doc]

**Supplementary Table S2**

**Primary Antibodies**

mAb: monoclonal antibody; pAb: polyclonal antibodies; m: mouse; rb: rabbit; gp: guinea pig

| **Antigen** | **approx. mol. mass (kDa)** | **Antibody type, species** | **Source** | | |
| --- | --- | --- | --- | --- | --- |
| **Transmembrane Proteins and Glycoproteins** | | | | | |
| **Adherens Junctions** | | | | | |
| E-Cadherin | 120 | mAb, m | | Transduction Laboratories (Lexington, KY, USA) | |
|  |  | mAb, m | | BD Biosciences (Heidelberg, Germany) | |
|  |  | pAb, rb | | Epitomics (Burlingame, CA, USA) | |
|  |  | mAb, rb | | Epitomics | |
| N-Cadherin | 130 | mAb, m | | Transduction Laboratories | |
|  |  | mAb, m | | BD Biosciences | |
|  |  | pAb, rb | | QED Biosciences (San Diego, CA, USA) | |
| P-Cadherin | 120 | mAb, m | | Transduction Laboratories | |
|  |  | mAb, m | | BD Biosciences | |
| VE-Cadherin | 135 | pAb, rb | | Cayman Chemical Company (Ann Arbor, MI, USA) | |
|  |  | mAb, m (BV9) | | Gift of E. Dejana (Univeristy of Milan, Italy) | |
|  |  | mAb, m (BV9) | | Progen Biotechnik (Heidelberg, Germany) | |
| Cadherin-11 | 120 | mAb, m | | Zymed (now Life Technologies; Darmstadt, Germany) | |
|  |  | mAb, m | | Invitrogen (now Life Technologies; Darmstadt, Germany) | |
|  |  | pAb, rb | | Zymed | |
| **Desmosomes** | | | | | |
| Desmoglein 1 | 165 | mAb (P23) | | Progen Biotechnik | |
| Desmoglein 2 | 165 | mAb, m (10G11) | | Progen Biotechnik | |
|  |  | mAb, m (G96) | | Progen Biotechnik | |
|  |  | mAb, m (G129) | | Progen Biotechnik | |
|  |  | mAb, m | | Zytomed Systems (Berlin, Germany) | |
|  |  | pAb, rb | | Progen Biotechnik | |
|  |  | pAb, gp | | Progen Biotechnik | |
|  | 122 | pAb, rb (rb 5) | | Progen Biotechnik | |
| Desmoglein 1+2 |  | mAb, m (DG3.10) | | Progen Biotechnik | |
| Desmoglein 3 | 140 | mAb, m (G194) | | Progen Biotechnik | |
| Desmoglein 4 | 108* | pAb, gp | | Progen Biotechnik | |
| Desmocollin 1 | 111 | mAb, m (U100) | | Progen Biotechnik | |
| Desmocollin 2 | 130 | pAb, rb (rb 36) | | Progen Biotechnik | |
|  |  | pAb, gp | | Progen Biotechnik | |
| Desmocollin 3 | 109 | mAb, m (U114) | | Progen Biotechnik | |
| * Polypeptide sequence only (Whittock and Bower 2003 J. Invest. Dermatol. 120, 523-530) | | | | | |
| **Antigen** | **approx. mol. mass (kDa)** | **Antibody type, species** | | | **Source** |
| **Transmembrane Proteins and Glycoproteins (cont'd)** | | | | | |
| **Tight Junction Proteins** | | | | | |
| Occludin | 65 | mAb, m | | | Invitrogen |
|  |  | mAb, m | | | Zymed |
|  |  | mAb, rat5 (MOC37) | | | Zymed |
|  |  | pAb, rb | | | Zymed |
| Claudin-1 | 22 | mAb, m | | | Invitrogen |
|  |  | pAB, rb | | | Zymed |
|  |  | pAB, rb | | | Invitrogen |
| Claudin-2 | 22 | mAb, m | | | Invitrogen |
|  |  | pAb, rb | | | Zymed |
| Claudin-3 | 22 | pAb, rb | | | Zymed |
| Claudin-4 | 22 | mAb, m | | | Zymed |
|  |  | pAb, rb | | | Invitrogen |
| Claudin-5 | 22 | mAb, m | | | Zymed |
| Claudin-11 | 27 | pAb, rb | | | Zymed |
| **Further Transmembrane Proteins** | | | | | |
| Protein PERP | 21 | mAb m (26.3.30) | | | Progen Biotechnik |
|  |  | mAb, m (8.2.9) | | | Progen Biotechnik |
|  |  | mAb, m (26.2.22) | | | Progen Biotechnik |
|  |  | pAb, gp  (PERP-1A-4B) | | | Progen Biotechnik |
| Epithelial Cell Adhesion Molecule (EpCAM) | 37, 40 | mAb, m (HEA125) | | | Progen Biotechnik |
|  |  | mAb, m (MOC-31) | | | Progen Biotechnik |
|  |  | mAb, m (33.2) | | | Gift of G. Moldenhauer, German Cancer Research Center, Heidelberg, Germany |
| **Plaque Proteins** | | | | | |
| α-Catenin | 102 | pAb, rb | | | Sigma-Aldrich  (Taufkirchen, Germany) |
|  |  | mAb, m | | | Zymed |
| β-Catenin | 102 | mAb, m | | | Transduction Laboratories |
|  |  | mAb, rb | | | Epitomics |
|  |  | pAb, rb | | | Sigma-Aldrich |
| Protein p120 | 135 | mAb, m | | | Transduction Laboratories |
|  |  | pAb, rb | | | Sigma-Aldrich |
| Protein p0071 | 51 | mAb, m  (SEPP 7.7.9) | | | Progen Biotechnik |
|  |  | pAb, gp (GP71) | | | Progen Biotechnik |
| Protein ARVCF | 120 | pAb, gp | | | Gift of I. Hofmann, German Cancer Research Center, Heidelberg, Germany |
| Plakoglobin | 82 | mAb, m  (PG 5.1.7.2) | | | Progen Biotechnik |
|  |  | mAb, m (PG 11E4) | | | Gift of M.J. Wheelock, University of Nebraska, Omaha, NE, USA |
| **Antigen** | **approx. mol. mass (kDa)** | **Antibody type, species** | | | **Source** |
| **Plaque Proteins (cont'd)** | | | | | |
| Plakophilin 1 | 80 | mAb, m (5C2) | | | Progen Biotechnik |
|  |  | mAb, m (2D6) | | | Progen Biotechnik |
| Plakophilin 2 | 97 | mAb, m  (Pkp 2-518) | | | Progen Biotechnik |
|  |  | mAb, m  (CM-62, -86, -150) | | | Progen Biotechnik |
| Plakophilin 2 | 97 | pAb, gp (SP-PP2) | | | Progen Biotechnik |
| Plakophilin 3 | 87 | mAb, m (270.6.2) | | | Progen Biotechnik |
|  |  | gp | | | Progen Biotechnik |
| Desmoplakin | 220 | mAb, m  (DP Epitope „Mix“ 2.15, 2.17, 2.20) | | | Progen Biotechnik |
|  |  | pAb, gp | | | Progen Biotechnik |
| Ezrin | 80 | mAb, m | | | Sigma Aldrich |
| Vinculin | 130 | mAb, m (11-5) | | | Sigma-Aldrich |
| α-Actinin | 102 | mAb, m (BM 75.2) | | | Sigma-Aldrich |
|  |  | mAb, m (EA-53) | | | Sigma-Aldrich |
|  |  | pAb, rb | | | Sigma Aldrich |
| l/s-Afadin | 190 | pAb, rb | | | Sigma-Aldrich |
| Protein ZO-1 | 195 | mAb, m | | | Zymed |
|  |  | pAb, rb | | | Zymed |
| Protein Myozap | 54 | mAb, m (517.67) | | | Progen Biotechnik |
|  |  | pAb, gp (2A) | | | Progen Biotechnik |
| Striatin | 110 | pAb, rb | | | Millipore (Billerica, MA, USA) |
|  |  | pAb, rb | | | Sigma |
|  |  | mAb, m | | | BD Transduction Laboratories |
| LUMA | 43 | mAb, m (E-1) | | | Santa Cruz Biotechnology (CA, USA) |
|  |  | pAb, gp | | | Franke et al. 2014 |
| **Microfilament and Intermediate-Sized Filament Proteins** | | | | | |
| Actin | 42 | mAb, m | | | Sigma-Aldrich |
| Actin (sarcomeric) |  | mAb, m | | | Sigma-Aldrich |
| β-Actin |  | mAb, m | | | Progen Biotechnik |
| Smooth muscleα-actin | | mAb, m (ASM-1) | | | Progen Biotechnik |
| Cardiac/embryonic α-actin | | mAb, m (AC1-20.4.2) | | | Progen Biotechnik |
| Vimentin | 54 | mAb, m (3B4) | | | Progen Biotechnik |
|  |  | pAb, gp  (bVim A+B 06/10) | | | Progen Biotechnik |
| Desmin | 53 | mAb, m (D9) | | | Progen Biotechnik |
| Neurofilament protein | 200 | mAb, m | | | Progen Biotechnik |
|  | 160 | mAb, m | | | Progen Biotechnik |
|  | 68 | mAb, m | | | Sigma-Aldrich |
|  |  | mAb, m | | | Roche (Mannheim, Germany) |
| Cytokeratin 8 | 53 | mAb, m (17.2) | | | Progen Biotechnik |
|  |  | pAb, gp (rec B 10/10) | | | Progen Biotechnik |
| Cytokeratin 18 | 48 | mAb, m (18.04 / 214) | | | Progen Biotechnik |
|  |  | pAb, gp (rec B 10/10) | | | Progen Biotechnik |
